# An ALS-associated mutation dysregulates microglia-derived extracellular microRNAs in a sex-specific manner

**DOI:** 10.1242/dmm.050638

**Published:** 2024-05-29

**Authors:** Eleni Christoforidou, Libby Moody, Greig Joilin, Fabio A. Simoes, David Gordon, Kevin Talbot, Majid Hafezparast

**Affiliations:** ^1^ Sussex Neuroscience, School of Life Sciences, University of Sussex, Brighton, BN1 9QG, UK; ^2^ Nuffield Department of Clinical Neurosciences, University of Oxford, Oxford, OX3 9DU, UK; ^3^ Kavli Institute for Nanoscience Discovery, University of Oxford, Oxford, OX1 3QU, UK

**Keywords:** Microglia, MicroRNA, TDP-43, Amyotrophic lateral sclerosis, Motor neuron disease

## Abstract

Evidence suggests the presence of microglial activation and microRNA (miRNA) dysregulation in amyotrophic lateral sclerosis (ALS), the most common form of adult motor neuron disease. However, few studies have investigated whether the miRNA dysregulation originates from microglia. Furthermore, TDP-43 (encoded by *TARDBP*), involved in miRNA biogenesis, aggregates in tissues of ∼98% of ALS cases. Thus, this study aimed to determine whether expression of the ALS-linked TDP-43^M337V^ mutation in a transgenic mouse model dysregulates microglia-derived miRNAs. RNA sequencing identified several dysregulated miRNAs released by transgenic microglia and a differential miRNA release by lipopolysaccharide-stimulated microglia, which was more pronounced in cells from female mice. We validated the downregulation of three candidate miRNAs, namely, miR-16-5p, miR-99a-5p and miR-191-5p, by reverse transcription quantitative polymerase chain reaction (RT-qPCR) and identified their predicted targets, which primarily include genes involved in neuronal development and function. These results suggest that altered TDP-43 function leads to changes in the miRNA population released by microglia, which may in turn be a source of the miRNA dysregulation observed in the disease. This has important implications for the role of neuroinflammation in ALS pathology and could provide potential therapeutic targets.

## Teaser

**Summary:** An ALS-associated mutation in TDP-43 changes the microglia-derived miRNA population, which may be the source of the miRNA dysregulation observed in the disease and potentially perturbed gene expression in neighbouring neurons.

## INTRODUCTION

Amyotrophic lateral sclerosis (ALS) is the most severe and most common form of motor neuron degeneration in adults, caused by the death of motor neurons in the motor cortex, brainstem and spinal cord. Although ALS has been traditionally considered as having cell-autonomous mechanisms (i.e. damage within the motor neurons being sufficient to cause disease), numerous lines of evidence suggest that the death of motor neurons is influenced by non-neuronal cells such as astrocytes and microglia (Boillée et al., 2006; Kang et al., 2013; Yamanaka and Komine, 2018; Vahsen et al., 2021), and non-cell-autonomous mechanisms appear to play significant roles in disease onset and/or progression. In fact, studies on superoxide dismutase 1 (SOD1) transgenic mouse models suggest that isolated expression of disease-associated protein variants in motor neurons are insufficient for disease onset (Lino et al., 2002; Pramatarova et al., 2001), which requires simultaneous expression within glial cells (Clement et al., 2003; Yamanaka et al., 2008). In line with this, there is further evidence that the expression of SOD1^G37R^ in motor neurons underlies disease onset and that reducing the expression of the mutant protein within microglia slows disease progression (Boillée et al., 2006). Importantly, increased numbers of activated microglia have been observed in the central nervous system (CNS) of ALS mouse models and patients (Hall et al., 1998; McGeer et al., 1993). Specifically, studies on post-mortem tissues have shown increased levels of activated microglia in areas of the brain with neuronal loss (Boillée et al., 2006; Polazzi and Monti, 2010). Most recently, microglia derived from induced pluripotent stem cells from patients with ALS carrying a *C9orf72* mutation have been shown to exert toxicity on motor neurons via pro-inflammatory pathways (Vahsen et al., 2023).

MicroRNAs (miRNAs) are small non-coding RNAs (ncRNAs) that primarily bind to complementary messenger RNA (mRNA) sequences, resulting in gene silencing via degradation or translational repression (Bartel, 2009), but can also interact with promoters to activate gene expression (Ramchandran and Chaluvally-Raghavan, 2017). Evidence for a role for miRNAs in ALS pathology comes from the observation of a differential miRNA expression profile between patients with ALS and unaffected individuals in circulating fluids, particularly in the cerebrospinal fluid and blood (Joilin et al., 2019, 2020, 2022; Benigni et al., 2016; De Felice et al., 2014; Freischmidt et al., 2013), giving rise to the opportunity of using these miRNAs as potential biomarkers (Joilin et al., 2019; Ricci et al., 2018). However, the source of these miRNAs is unknown and it is unclear whether these are released by atrophied muscles, degenerating motor neurons or other cell types such as the glia. In addition, FUS and TDP-43, proteins associated with ALS, are directly involved in miRNA processing (Morlando et al., 2012; Kawahara and Mieda-Sato, 2012). Consequently, their mislocalisation in cytoplasmic aggregates in ALS may be associated with the miRNA dysregulation observed in patients with ALS.

A recently described transgenic mouse model carrying the human *TARDBP* gene (encoding TDP-43) with the ALS-associated M337V mutation has provided new insights into the mechanisms of TDP-43 pathology (Gordon et al., 2019). This mouse model expresses a single copy of the gene integrated into a defined neutral position within the mouse genome. Expression of this mutant transgene results in a progressive motor deficit, a loss of neuromuscular junction integrity and a decrease in survival. Furthermore, primary motor neurons from this model exhibit a defect in stress granule dynamics and TDP-43 mislocalisation. Given the known role of TDP-43 in RNA metabolism and miRNA processing, this model is useful in facilitating the investigation of altered miRNA processes in ALS.

In microglia, TDP-43 pathology disrupts normal functioning, including impaired phagocytosis, altered cytokine production and aberrant activation states, which contribute to neurodegenerative processes in ALS. A study demonstrated that monocyte-derived microglia-like cells from patients with ALS exhibited TDP-43-positive inclusions, significantly impaired phagocytosis, altered cytokine profiles, and abnormal morphologies suggesting a neuroinflammatory phenotype, which are consistent with disease progression (Quek et al., 2022). Furthermore, TDP-43 depletion in microglia has been shown to promote amyloid clearance but also to induce synapse loss, suggesting a complex role in neurodegenerative processes (Paolicelli et al., 2017). These findings suggest that TDP-43 dysfunction in microglia is a potential driving force in the pathogenesis of ALS.

The M337V mutation in TDP-43 is associated with alterations in protein-protein interactions and stress granule dynamics in motor neurons, impacting the response to oxidative stress. These alterations can lead to progressive motor dysfunction (Gordon et al., 2019). In terms of its influence on cellular processes, the M337V mutation affects the conformational properties of the TDP-43 protein, resulting in an impairment in the phase separation ability of the protein and an increased tendency towards fibril formation, which is significant in ALS pathology (Zeng et al., 2023). Furthermore, studies using mouse models have shown that overexpression of human *TARDBP^M337V^* leads to worse disease features compared to those in mice expressing wildtype human *TARDBP*. These include increased gliosis, accumulation of ubiquitinated proteins and alterations in mitochondrial structures in neurons, which are indicative of a more severe neurodegenerative process (Janssens et al., 2013). Lastly, research involving allele-specific knockdown of the M337V mutant allele in neural stem cells derived from induced pluripotent stem cells has shown that targeting this specific mutation can reduce cytoplasmic inclusions in cells. This suggests potential therapeutic avenues for ALS cases involving this mutation (Nishimura et al., 2014). These studies collectively highlight the unique aspects of the M337V mutation in TDP-43 and its role in ALS pathology.

We previously reviewed the evidence implicating reactive microglia and dysregulated miRNAs in ALS, and explored how microglia may potentially be the source of this miRNA dysregulation (Christoforidou et al., 2020). We concluded that this is likely because microglia release extracellular vesicles containing miRNAs, which play a role in gene regulation, and alterations in these miRNAs along with inflammation and changes in microglial phenotypes are observed in patients with ALS. ​Based on these observations, we hypothesised that expression of the ALS-linked TDP-43^M337V^ mutation dysregulates microglia-derived miRNAs and that this may further be affected by their activation state. To test this hypothesis, here, we used next-generation sequencing to profile and interrogate the comparative expression of miRNAs released by transgenic and non-transgenic microglia, in the presence or absence of the pro-inflammatory stimulus lipopolysaccharide (LPS). Given the well-documented sex differences in the prevalence and incidence of ALS (Manjaly et al., 2010; McCombe and Henderson, 2010) and in line with recommendations from the European Commission's Horizon 2020 framework and the UK's National Centre for the Replacement, Refinement and Reduction of Animals in Research (NC3Rs), which advocate for considering sex as a biological variable in biomedical research, we ensured the inclusion of both males and females in this study to account for the potential impact of sex on any observed effects.

Here, we found that the *TARDBP^M337V^* transgene did not significantly affect the microglial response to LPS in terms of TNF and IL-1β (encoded by *Il1b*) cytokine induction. However, the transgene appeared to influence miRNA release in a sex-dependent manner, with female microglia showing a more pronounced effect. We identified a total of 391 miRNAs, with males and females showing distinct profiles of upregulated and downregulated miRNAs in response to LPS treatment. Furthermore, genotype significantly impacted miRNA release only in female samples, implying that the presence of the *TARDBP* transgene influences miRNA release, particularly in females. Notably, this effect appeared to be dose dependent, with greater miRNA dysregulation in homozygous lines. These findings reveal a complex interplay among genotype, treatment response and sex in determining the miRNA profile released from microglia, potentially influencing distinct mechanisms of neuroinflammation.

## RESULTS

### Impact of the *TARDBP^M337V^* transgene on the microglial cytokine response to LPS

**Fig. 1.**
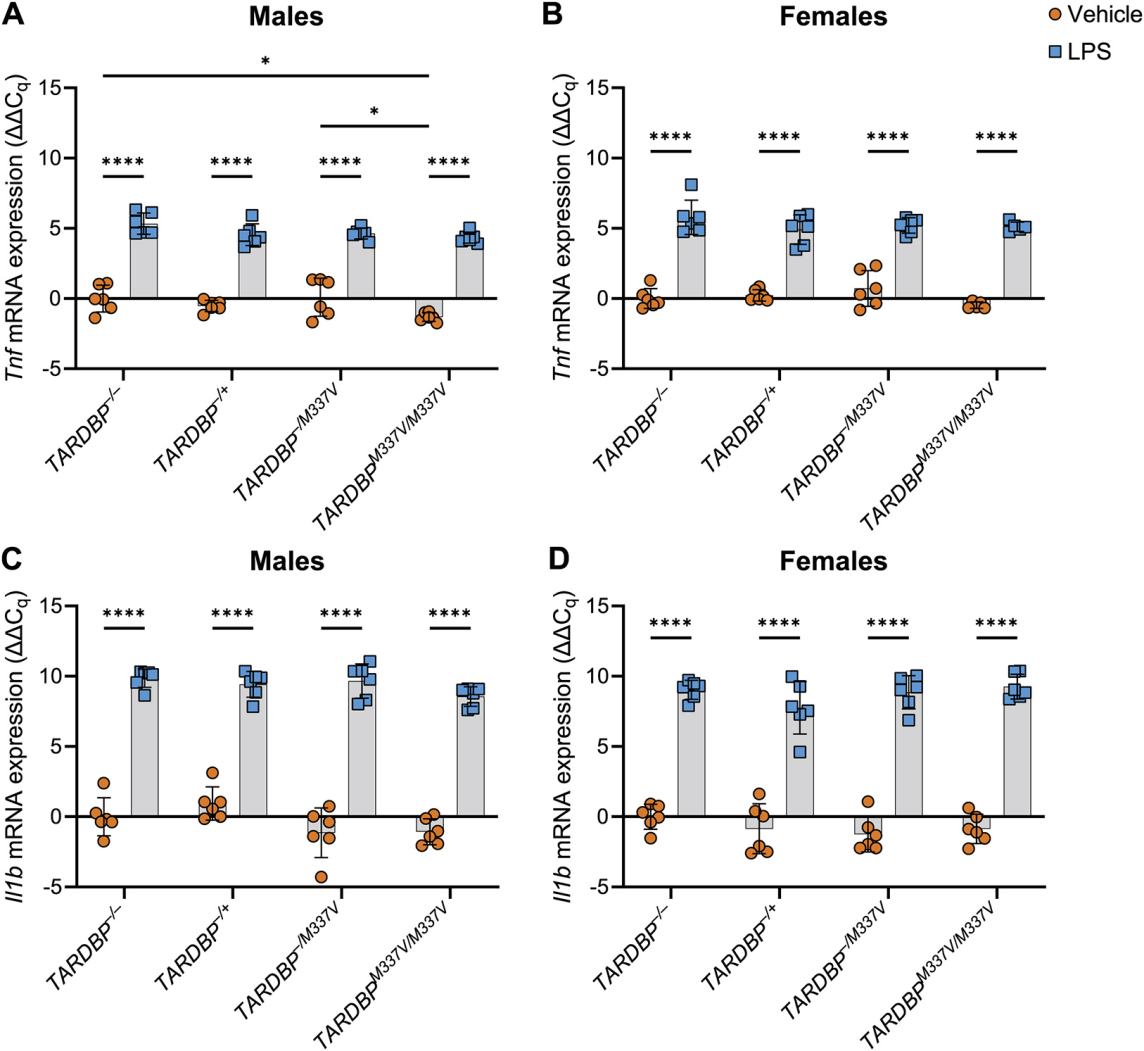
***Tnf* and *Il1b* expression is induced within microglia upon LPS treatment.** (A,B) *Tnf* mRNA expression. One outlier was removed from the female *TARDBP^M337V/M337V^* data. (C,D) *Il1b* mRNA expression. Following normalisation to housekeeping genes (*Gapdh* and *Pgk1*), expression values (ΔC_q_) were normalised to the mean of values from the vehicle-treated samples from *TARDBP^−/−^* animals within each sex group to obtain ΔΔC_q_ values [i.e. ΔΔC_q_ values were calculated as (vehicle-treated *TARDBP^−/−^* ΔC_q_ mean) minus (individual ΔC_q_ value)]. This normalisation process resulted in some vehicle sample values being slightly above or below zero. Data are shown as mean±s.d. *n*=6 biological replicates per genotype per sex. Two-way ANOVA with Šidák's post-hoc was used. Main effect of treatment (A-D): *P*<0.0001. Main effect of genotype (A): *P*=0.0090. Genotype×treatment interaction (D): *P*=0.0254. Pairwise comparisons: *P<0.05; ****P<0.0001.

Primary mouse microglia from neonatal transgenic (*TARDBP^−/+^*, *TARDBP^−/M337V^* and *TARDBP^M337V/M337V^*) and non-transgenic (*TARDBP^−/−^*) mice of both sexes were stimulated with 250 ng ml^−1^ LPS or vehicle for 24 h before the culture medium and cells were collected. To confirm the successful induction of a response in the cells by the LPS stimulus, gene expression of the pro-inflammatory cytokines TNF and IL-1β was quantified by reverse transcription quantitative polymerase chain reaction (RT-qPCR) as a robust and empirically validated approach to assess microglial response to LPS (Brás et al., 2020; Zhang et al., 2022; Ye et al., 2020). We used a repeated-measures two-way ANOVA for this analysis because each mouse (biological replicate) provided two cell culture wells of microglia: one treated with LPS and the other with vehicle. This approach enabled a paired comparison within each biological replicate, allowing us to assess the treatment effect while accounting for the biological variability present among different mice. As expected (Diz-Chaves et al., 2012; Kang et al., 2019), there was a significant increase in the expression of both *Tnf* and *Il1b* in all the samples following LPS treatment compared to their expression levels following vehicle treatment regardless of genotype (Fig. 1A-D), indicating a successful pro-inflammatory response after 24 h (main effect of treatment, *P*<0.0001 for both sexes). Additionally, in female samples, the baseline expression of *Tnf* and *Il1b* in the absence of LPS stimulation (i.e. only vehicle treatment) was not significantly different among cells of different genotype, suggesting that the presence of the human transgene does not alter the baseline expression levels of these pro-inflammatory cytokines (Fig. 1A-D; main effect of genotype, *P*≥0.05). However, in males, there was a significant main effect of genotype for *Tnf* only (*P*=0.0090). Post hoc analysis revealed this to be not only due to a significant downregulation of *Tnf* in vehicle-treated *TARDBP^M337V/M337V^* samples compared to its expression in *TARDBP^−/M337V^* samples (*P*=0.0143; Fig. 1A), indicating a potential influence of transgene copy number, but also due to a significant downregulation of *Tnf* in *TARDBP^M337V/M337V^* samples compared to its expression in *TARDBP^−/−^* samples (*P*=0.0251; Fig. 1A), indicating an effect of the mutation itself. Finally, for *Tnf*, there was no significant interaction between treatment and genotype in either sex. However, for *Il1b*, there was a statistically significant (*P*=0.0254) interaction between treatment and genotype in females only. Samples from individual mice (female only) also exhibited significant variation (*P*<0.0001) in their expression of *Il1b*.

In summary, these data demonstrate that although the *TARDBP^M337V^* transgene might influence the baseline expression levels of *Tnf* in male microglia, it does not appear to affect the overall response of microglia to LPS stimulation. However, the significant interaction between treatment and genotype in female microglia for *Il1b* indicates that the relationship between transgene presence and cytokine response may be more complex and warrants further investigation. These findings provide an important foundation for understanding how TDP-43 mutations may modulate microglial activation and inflammatory response in the context of ALS.

### Quality of sequencing and alignment information

**Fig. 2.**
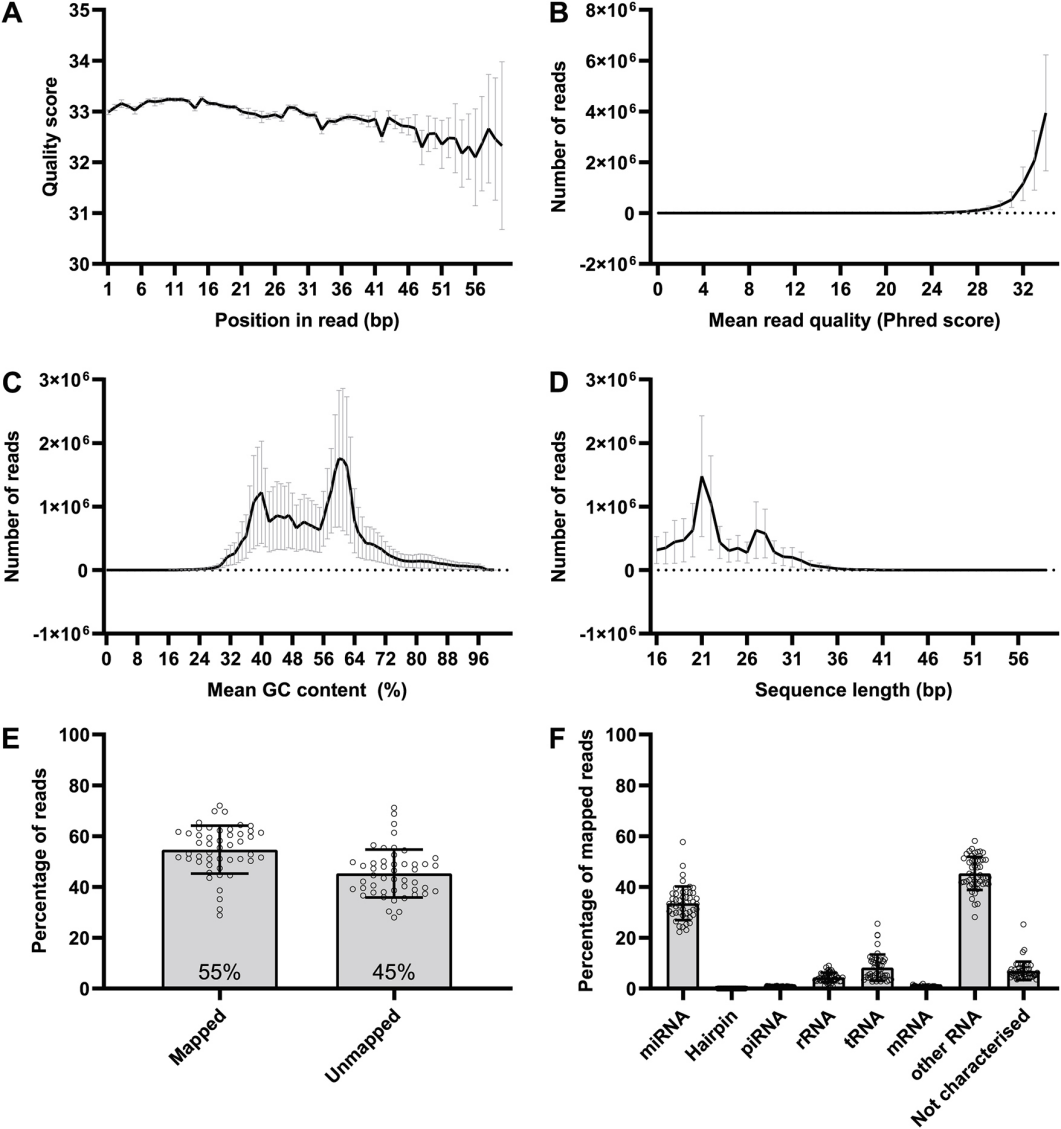
**Quality control of sequencing reads and mapping information.** (A) Per-base sequence quality. (B) Per-sequence quality scores. (C) Per-sequence GC content. (D) Sequence length distribution. (E) Percentage of mapped and unmapped reads in each sample sequenced (mean percentage value is indicated within the bars). (F) Percentage of mapped reads. ‘Not characterised’ reads are those that aligned to the genome but in a location that does not correspond to currently known RNA sequences. Data are shown as mean±s.d. *n*=48 samples.

Following exposure of microglia to LPS or vehicle for 24 h, the culture medium was collected and the total RNA released by the cells was extracted. This includes small ncRNAs such as miRNAs released by the microglia during the 24-h treatment period. This extracellular RNA, which may contain protein-bound miRNAs as well as miRNAs enclosed in extracellular vesicles, was used to prepare miRNA libraries for next-generation sequencing. Quality control of the sequencing data revealed that all the per-base sequence qualities (Fig. 2A) and per-sequence quality scores (Fig. 2B) were above the threshold of 28, indicating that the sequencing results of all samples were of good quality. Moreover, the average GC content per read was 54% (Fig. 2C) and, as expected, most reads were ∼22 bp in size (corresponding to the average size of miRNAs) and within the ∼24-30 bp range [corresponding to the size range of PIWI-interacting RNA (piRNA); Fig. 2D]. Of the total sequencing reads in each sample, 55% on average mapped to the mouse genome (Fig. 2E) and most of the remaining reads did not map due to being too short (Fig. S3). Mapped reads mainly consisted of miRNA (33.58%) and ‘other’ RNA (45.32%), which included long non-coding RNA (lncRNA), small nuclear RNA, small nucleolar RNA and predicted RNA sequences (Fig. 2F). The inclusion of lncRNAs in small RNA sequencing is likely because lncRNAs can be processed into smaller fragments, which then fall within the size range targeted by small RNA sequencing protocols. Other constituents of the mapped reads were transfer RNAs (tRNAs, 8.25%), ribosomal RNAs (rRNAs, 4.40%), piRNAs (0.72%), mRNAs (0.70%), hairpin RNAs (0.05%), and ‘not characterised’ reads (6.98%) that aligned to locations of the genome that do not correspond to currently known RNA sequences (Fig. 2F).

### The *TARDBP^M337V^* transgene dysregulates miRNAs released from microglia in a sex-specific manner

**Fig. 3.**
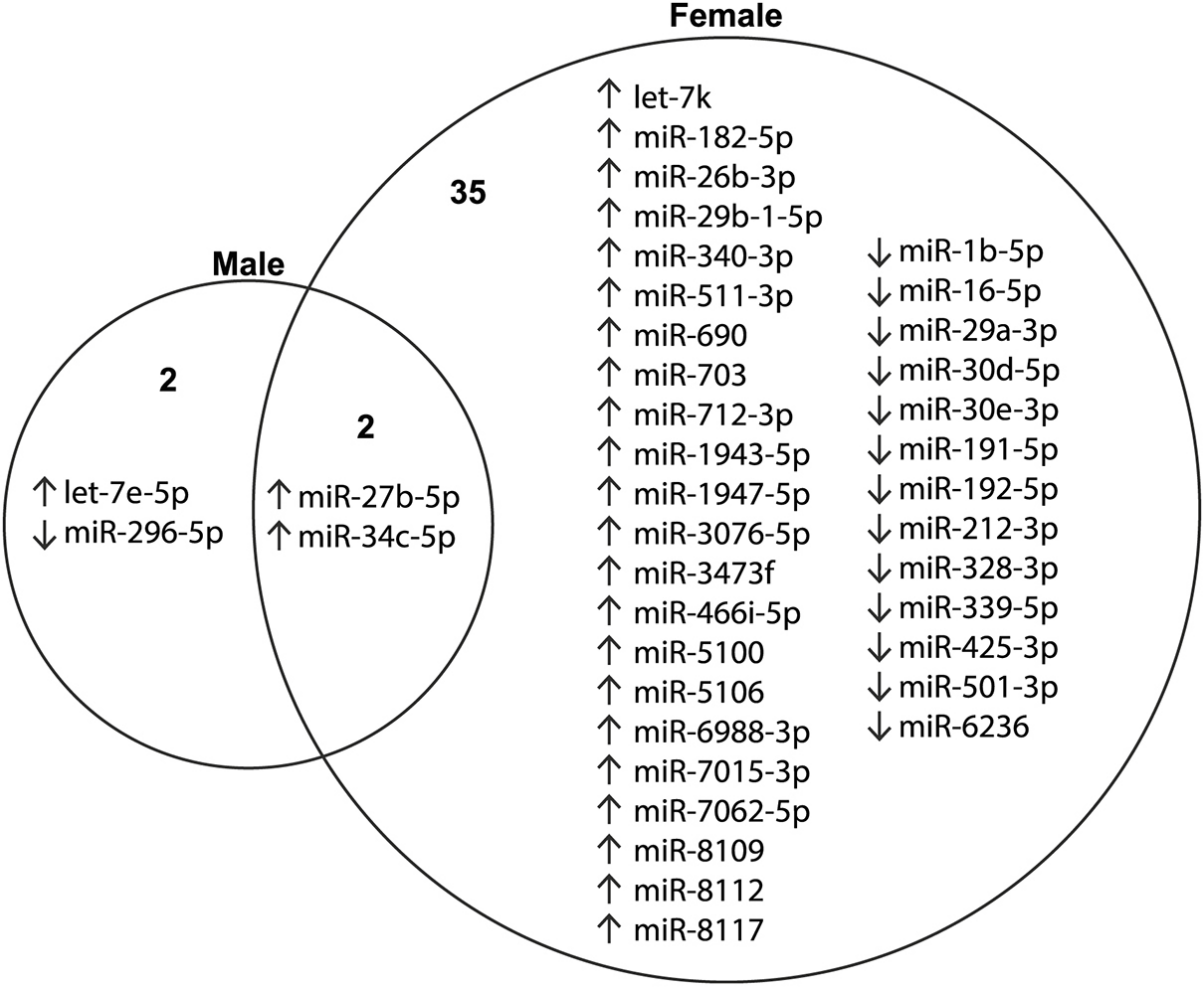
**miRNAs with dysregulated release into the culture medium upon LPS stimulation, after adjusting for genotype effects.** ‘Up’ arrows indicate upregulation and ‘down’ arrows indicate downregulation of miRNA levels in the culture medium following LPS stimulation, compared to those for vehicle treatment. *n*=3 biological replicates per genotype per sex. A list of these dysregulated miRNAs and associated significance values is also provided in Table S1.

A total of 391 miRNAs were detected in at least one of the 48 miRNA libraries (made from RNA released by microglia in the culture media) that were sequenced. The differential expression analysis of these miRNAs was undertaken separately for males and females. In male samples, after controlling for the genotype effect, we identified three upregulated miRNAs and one downregulated miRNA upon LPS treatment, compared to vehicle treatment. In contrast, in female samples we identified 22 upregulated and 13 downregulated miRNAs (Fig. 3; Table S1), only two of which (miR-27b-5p and miR-34c-5p) were common with those identified in males. This stark disparity underscores a potent sex-specific effect on the microglial response to LPS.

**Fig. 4.**
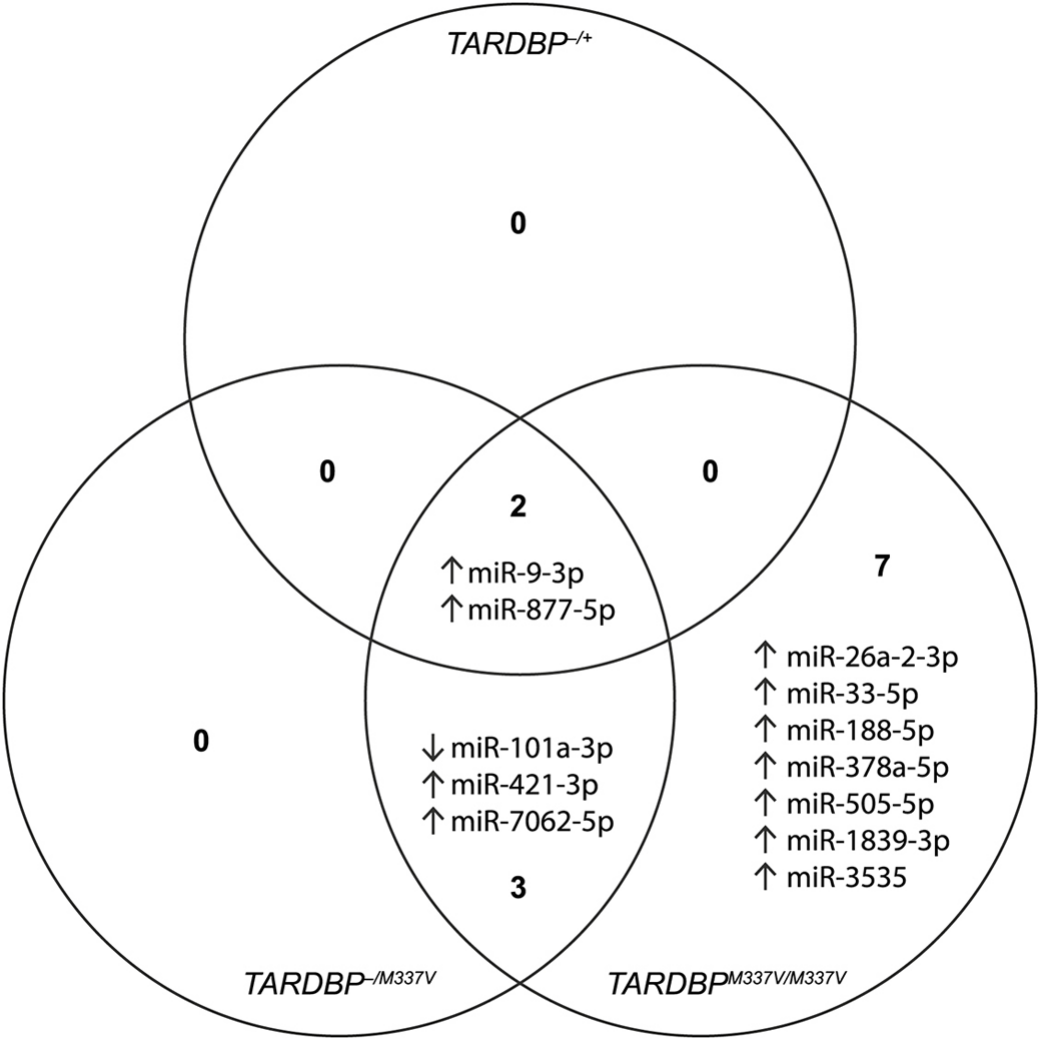
**miRNAs with dysregulated release into the culture medium in female transgenic samples, after adjusting for treatment effects.** ‘Up’ arrows indicate upregulation and ‘down’ arrows indicate downregulation of miRNA levels for the indicated genotype, compared to those for non-transgenic controls. *n*=3 biological replicates per genotype per sex. A list of these dysregulated miRNAs and associated significance values is also provided in Table S2.

To assess the impact of human transgene expression on extracellular miRNA release from microglia, after adjusting for treatment effects, we compared each transgenic group with the non-transgenic controls, stratified by sex. Notably, genotype significantly affected miRNA release only in female samples (Fig. 4; Table S2). Specifically, we identified two upregulated miRNAs in *TARDBP^−/+^* samples, five in *TARDBP^−/M337V^* samples and 12 in *TARDBP^M337V/M337V^* samples, compared to non-transgenic controls. Interestingly, the two upregulated miRNAs (miR-9-3p and miR-877-5p) in *TARDBP^−/+^* samples were also found to be upregulated in *TARDBP^−/M337V^* and *TARDBP^M337V/M337V^* samples. The recurrence of these two dysregulated miRNAs across all transgenic genotypes suggests that the presence of the human *TARDBP* transgene, irrespective of its mutational status, has a baseline influence. However, we observed a heightened severity in the dysregulation of these two miRNAs in the two mutant transgenic groups compared to the wildtype transgenic group (Table 1). This pattern suggests that the TDP-43^M337V^ mutation not only determines the subset of affected miRNAs, but also intensifies the degree of their dysregulation, underscoring the exacerbating role of the mutation in perturbing miRNA expression profiles. Furthermore, these two miRNAs showed higher dysregulation in homozygous than in hemizygous mutant transgenics (Table 1) and, similarly, there were an additional three miRNAs (miR-101a-3p, miR-421-3p and miR-7062-5p) commonly dysregulated between *TARDBP^−/M337V^* and *TARDBP^M337V/M337V^* samples, but with a higher dysregulation in homozygous than in hemizygous mutant transgenics (Fig. 4; Table 1). This suggests a dose-dependent effect of the TDP-43 mutation on miRNA release, with higher mutation levels associated with a greater degree of miRNA dysregulation. Overall, the minimal miRNA dysregulation observed in the female wildtype transgenic samples suggests that the presence of the wildtype human *TARDBP* transgene exerts a limited impact on miRNA release from microglia, whereas the mutant *TARDBP* transgene exerts a larger effect.

**Table 1.**
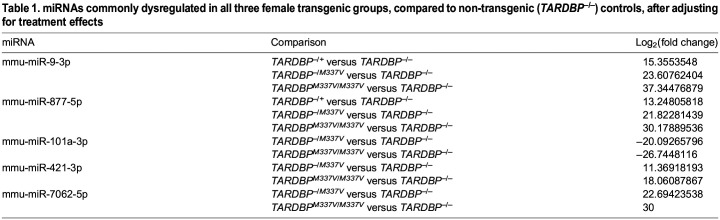
miRNAs commonly dysregulated in all three female transgenic groups, compared to non-transgenic (*TARDBP^–/–^*) controls, after adjusting for treatment effects

**Fig. 5.**
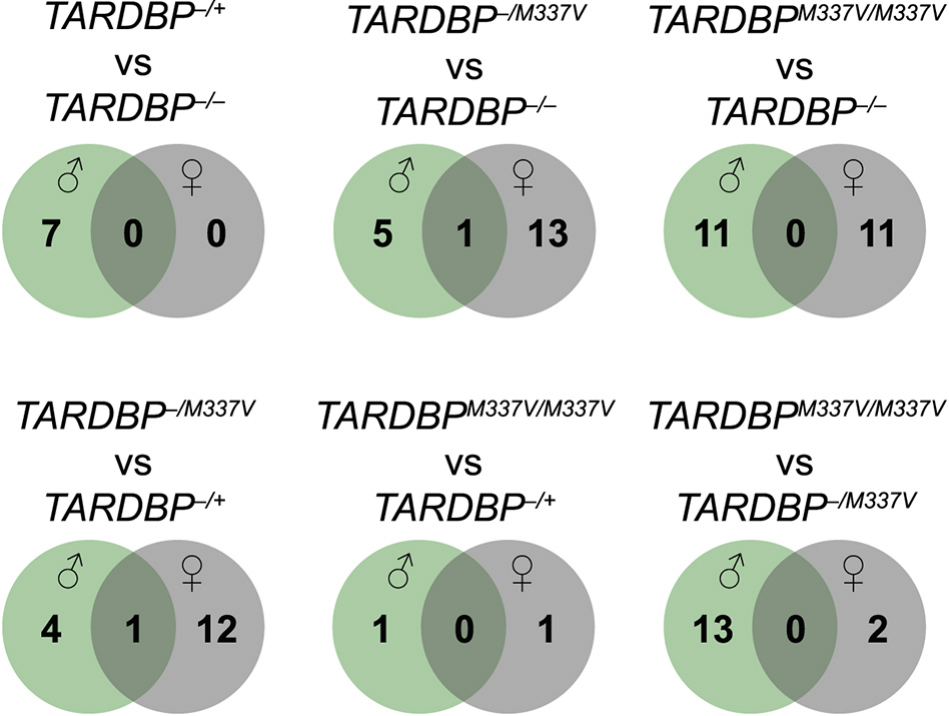
**Comparison of miRNAs with dysregulated release into the culture medium upon LPS treatment between male and female samples.** *n*=3 biological replicates per genotype per sex. A list of the dysregulated miRNAs and associated significance values is provided in Table S3.

Next, in order to discern how miRNA release following stimulation with LPS differs between genotypes, we examined the interaction between genotype and treatment. We conducted this analysis separately for each sex. Our findings revealed statistically significant miRNA dysregulation for most comparisons and for both sexes (Table S3). There were significant differences in the response to LPS treatment (compared to that following vehicle treatment), with little overlap between different genotypes (Table S4). Importantly, for each pairwise genotype comparison, different miRNAs were dysregulated in males than in females, with minimal overlap between the sexes (Fig. 5). Similar to the genotype-only effects discussed earlier, when juxtaposing the homozygous mutant transgenics against the hemizygous wildtype transgenics, there was a relatively modest degree of dysregulation (one dysregulated miRNA in each sex). Conversely, the comparison of the homozygous mutant transgenics to the non-transgenics showcased a greater number of dysregulated miRNAs (11 dysregulated miRNAs in each sex; Fig. 5). Given this pronounced effect, our subsequent RT-qPCR validation was directed towards the miRNAs identified as dysregulated in the comparison between homozygous mutant transgenics and non-transgenics. In this way, we aimed to provide a deeper insight into the most consequential alterations in miRNA profiles, underpinned by the presence of the TDP-43 mutation.

Overall, our findings underscore the differential response of male and female microglia to LPS treatment and demonstrate the significant impact of the presence and dose of the TDP-43 mutation on miRNA dysregulation, particularly in female microglia. These disparities in miRNA release profiles could potentially drive distinct molecular mechanisms of neuroinflammation in males and females, as well as in different genotypes.

### Validation of sequencing results by RT-qPCR

Next, we used RT-qPCR on RNA isolated from microglia-conditioned culture media to quantify the levels of four selected candidate miRNAs that we identified by next-generation sequencing as being dysregulated. As one of our main aims was to investigate the effect of LPS treatment in combination with the transgene effect, we selected for validation miRNAs that exhibited a statistically significant interaction between genotype and treatment. These included miR-16-5p and miR-191-5p, which showed significant interaction only in female samples, as well as miR-29b-3p and miR-99a-5p, which showed significant interaction only in male samples. The selection of these miRNAs for further analysis by RT-qPCR was based on their degree of dysregulation (greater than 5-fold change), availability of TaqMan Advanced miRNA assays and expression abundance (>2000 unique molecular identifiers) to ensure detection sensitivity, experimental reproducibility and biological significance. As with the sequencing data, the RT-qPCR data were analysed separately for males and females to ensure that our analysis was sensitive to sex-specific differences that could influence the outcome measures. To increase the statistical power of this analysis, we included three additional samples per genotype and per sex together with the original sequenced samples.

**Fig. 6.**
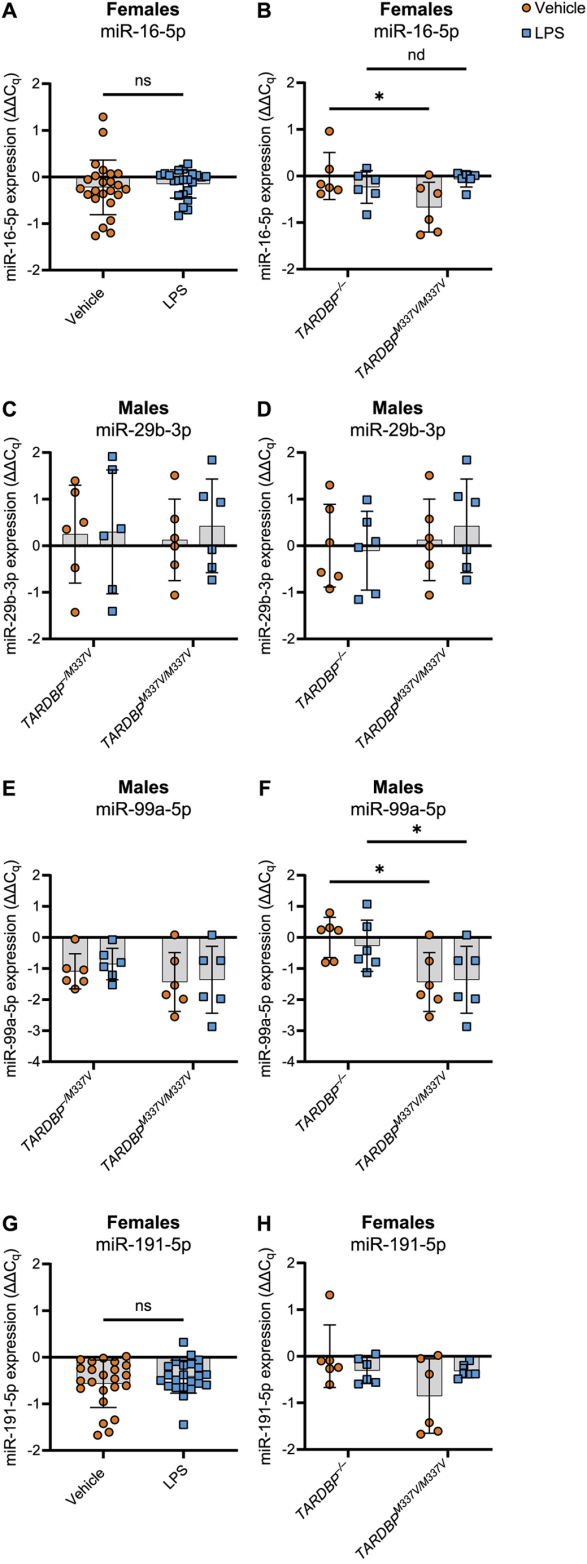
**RT-qPCR analysis confirms the dysregulated release of miRNAs from microglia into the culture medium as identified by sequencing.** Expression values (ΔΔC_q_) were normalised to the geometric mean of the miRNA expression for the vehicle-treated samples from *TARDBP^−/−^* animals within each sex group, following normalisation to the three least variable miRNAs identified by geNorm (miR-28a-3p, miR-32-5p and miR-190a-5p). Data are shown as mean±s.d. (A) Expression of miR-16-5p in female samples (all genotypes pooled). (B) Expression of miR-16-5p in female *TARDBP^−/−^* and *TARDBP^M337V/M337V^* samples. Significant genotype×treatment interaction: *P*=0.0206. (C) Expression of miR-29b-3p in male *TARDBP^−/M337V^* and *TARDBP^M337V/M337V^* samples. No significant main effects. (D) Expression of miR-29b-3p in male *TARDBP^−/−^* and *TARDBP^M337V/M337V^* samples. No significant main effects. (E) Expression of miR-99a-5p in male *TARDBP^−/M337V^* and *TARDBP^M337V/M337V^* samples. No significant main effects. (F) Expression of miR-99a-5p in male *TARDBP^−/−^* and *TARDBP^M337V/M337V^* samples. Significant main effect of genotype: *P*=0.0250. (G) Expression of miR-191-5p in female samples (all genotypes pooled). (H) Expression of miR-29b-3p in female *TARDBP^−/−^* and *TARDBP^M337V/M337V^* samples. No significant main effects. *n*=24 (A,G) or 6 (B-F,H) biological replicates per genotype. ns, not significant, *P*≥0.05; nd, no discovery (FDR≥0.05); * indicates discovery (FDR<0.05). Statistical tests: paired two-tailed *t*-test (A,G); repeated-measures two-way ANOVA (C-E,G); repeated-measures two-way ANOVA with two-stage step-up post hoc test (B,F);

**Fig. 7.**
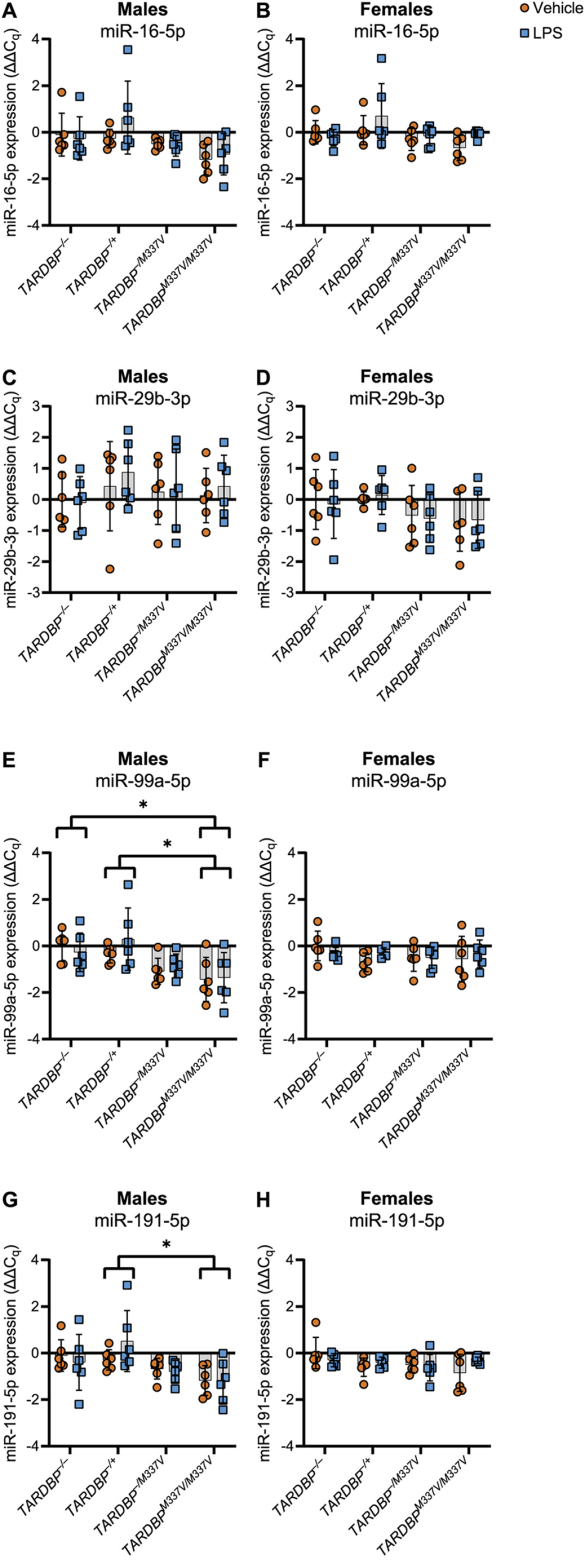
**RT-qPCR analysis of miRNAs released from microglia into the culture medium for all genotypes.** Expression values (ΔΔC_q_) are normalised to the geometric mean of the miRNA expression for the vehicle-treated samples from *TARDBP^−/−^* animals within each sex group, following normalisation to the three least variable miRNAs identified by geNorm (miR-28a-3p, miR-32-5p and miR-190a-5p). Data are shown as mean±s.d. (A,B) Expression of miR-16-5p in male (A) and female (B) samples. No significant main effects. (C,D) Expression of miR-29b-3p in male (C) and female (D) samples. No significant main effects. (E,F) Expression of miR-99a-5p in male (E) and female (F) samples. Males (E): significant main effect of genotype (*P*=0.0116). Females (F): no significant main effects. (G,H) Expression of miR-191-5p in male (G) and female (H) samples. Males (G): significant main effect of genotype (*P*=0.0192). Females (H): no significant main effects. *n*=6 biological replicates per genotype paired across treatments. *FDR<0.05. Statistical tests: repeated measures two-way ANOVA (A-D); repeated-measures two-way ANOVA with two-stage step-up post-hoc (E,G); mixed-effects model (due to one outlier removed from *TARDBP^−/+^*) (F,H).

For miR-16-5p, the sequencing data indicated a significant effect of LPS treatment compared to that of vehicle treatment in female, but not male, samples, after adjusting for the genotype effects (Table 2, comparison 1). Additionally, the sequencing data revealed a significant difference in the treatment response (i.e. difference in miR-16-5p expression between LPS and vehicle treatments) between female *TARDBP^M337V/M337V^* and female non-transgenic samples (Table 2, comparison 2). The normalised counts are shown in Fig. S1A,B. We did not see validation of the treatment effect of miR-16-5p in the RT-qPCR data (Table 3; Fig. 6A). However, we validated the significant interaction between treatment and genotype (*P*=0.0206) for this miRNA (Table 4). Post hoc analysis of the simple genotype effects within each treatment level revealed this difference to be significant specifically between vehicle-treated *TARDBP^M337V/M337V^* and vehicle-treated *TARDBP^−/−^* samples [false discovery rate (FDR)=0.0119], but not between LPS-treated samples of the same genotypes (Table 4; Fig. 6B). When we included all genotypes in statistical analysis, we did not observe any significant differences for this miRNA (Fig. 7A,B), suggesting a loss of statistical power.

**Table 2.**
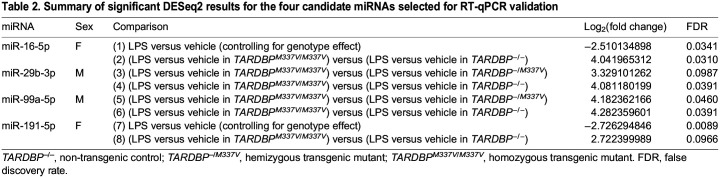
Summary of significant DESeq2 results for the four candidate miRNAs selected for RT-qPCR validation

**Table 3.**
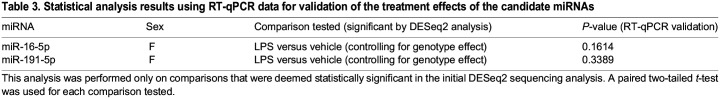
Statistical analysis results using RT-qPCR data for validation of the treatment effects of the candidate miRNAs

**Table 4.**
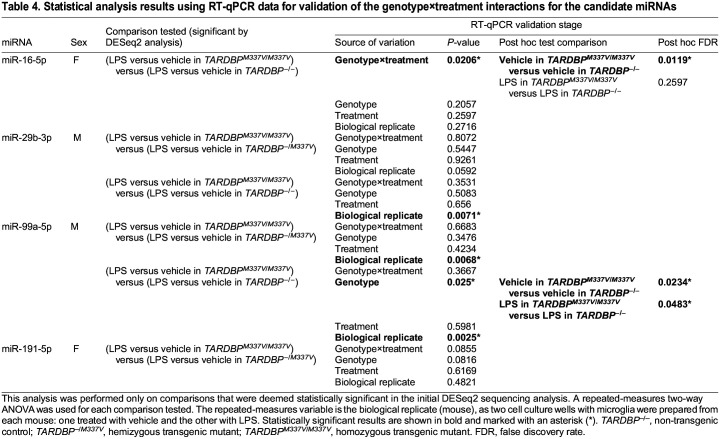
Statistical analysis results using RT-qPCR data for validation of the genotype×treatment interactions for the candidate miRNAs

For miR-29b-3p, the sequencing data showed a significant interaction between genotype and treatment when comparing male *TARDBP^M337V/M337V^* to male *TARDBP^−/M337V^* samples (Table 2, comparison 3), and when comparing male *TARDBP^M337V/M337V^* to male *TARDBP^−/−^* samples (Table 2, comparison 4). The normalised counts are shown in Fig. S1C,D. We did not see validation of these findings for miR-29b-3p in the RT-qPCR data (Table 4; Fig. 6C,D). However, we found a statistically significant variation in miR-29b-3p expression among samples originating from different biological replicates (*P*=0.0071; Table 4). This might have overshadowed any subtle genotype or treatment effects in the RT-qPCR data. This lack of significant differences persisted when all genotypes were included in the statistical analysis (Fig. 7C,D).

For miR-99a-5p, the sequencing results identified a statistically significant difference in the treatment response between male *TARDBP^M337V/M337V^* and male *TARDBP^−/M337V^* samples (Table 2, comparison 5), as well as between male *TARDBP^M337V/M337V^* and male non-transgenic samples (Table 2, comparison 6). The normalised counts are shown in Fig. S1E,F. Analysis of the RT-qPCR data did not validate the differences between *TARDBP^M337V/M337V^* and *TARDBP^−/M337V^* samples (Table 4; Fig. 6E). However, we found a statistically significant main effect of genotype (*P*=0.0250; Table 4). Post hoc analysis of the simple genotype effects within each treatment level revealed this difference to be significant specifically between vehicle-treated male *TARDBP^M337V/M337V^* and vehicle-treated male *TARDBP^−/−^* samples (FDR=0.0234), but also between LPS-treated male *TARDBP^M337V/M337V^* and LPS-treated male *TARDBP^−/−^* samples (FDR=0.0483), thus partially validating our sequencing findings (Table 4; Fig. 6F). However, there was also a significant variation in miR-99a-5p expression among biological replicates (*P*=0.0025; Table 4). This means that, even within the same genotype and treatment groups, samples from individual mice showed unique miR-99a-5p expression patterns. Inclusion of all genotypes in the statistical analysis revealed differences also between the *TARDBP^M337V/M337V^* and *TARDBP^−/+^* male, but not female, samples (Fig. 7E,F).

For miR-191-5p, the sequencing data indicated a significant effect of LPS treatment compared to that of vehicle treatment in female, but not male, samples averaged across all genotypes (Table 2, comparison 7). Additionally, the sequencing data revealed a statistically significant difference in the treatment response between female *TARDBP^M337V/M337V^* and female non-transgenic samples (Table 2, comparison 8). The normalised counts are shown in Fig. S1G,H. Analysis of the RT-qPCR data did not validate any of these findings for miR-191-5p (Tables 3 and 4; Fig. 6G,H). However, inclusion of all genotypes in the statistical analysis revealed differences between the *TARDBP^M337V/M337V^* and *TARDBP^−/+^* male, but not female, samples (Fig. 7G,H).

Overall, our RT-qPCR analysis identified miR-16-5p, miR-99a-5p and miR-191-5p as miRNAs that exhibit significant differential release by microglia. These findings partially validate our sequencing data using additional independent samples.

### Target prediction and gene set enrichment analysis

We used two prediction algorithms, TargetScan (Agarwal et al., 2015) and miRDB (Chen and Wang, 2020; Liu and Wang, 2019), to predict the genes that our three RT-qPCR-validated dysregulated miRNAs (miR-16-5p, miR-99a-5p and miR-191-5p) are likely to target. This analysis revealed 998 predicted targets for miR-16-5p, 59 for miR-99a-5p and 103 for miR-191-5p, combined from both algorithms. Of these, only 89 (8.9%) of the targets of miR-16-5p, 19 (32.2%) of the targets of miR-99a-5p and 25 (24.3%) of the targets of miR-191-5p were predicted by both algorithms (Table S5). The intersection of targets identified by both tools likely represents a higher-confidence subset of miRNA targets, as these genes were independently selected by distinct computational methodologies.

Following our predictive analysis, we expanded our investigation by consulting two online databases, miRTargetLink (Kern et al., 2021) and miRTarBase (Huang et al., 2022), to identify experimentally validated mRNA targets of miR-16-5p, miR-99a-5p and miR-191-5p. This approach not only allowed us to verify some of our predicted interactions, but also uncovered additional validated targets not predicted by our initial analysis, providing a more comprehensive view of the regulatory roles of these three miRNAs. This analysis revealed 53 validated targets for miR-16-5p, five for miR-99a-5p and three for miR-191-5p, combined from both databases. Of these, 43 (81.1%) of the targets of miR-16-5p and all (100%) of the targets of miR-99a-5p and miR-191-5p were listed in both databases (Table S6).

We subsequently used the intersection list of predicted targets with the intersection list of validated targets (excluding duplicates) as input to a gene set enrichment analysis (Gene Ontology analysis) to determine whether any biological processes were overrepresented in the list of these genes. We found that these gene targets were significantly enriched in biological processes critical for neuronal development, inflammatory responses and cell survival pathways (Table S7), highlighting their potential roles in the pathogenesis of ALS. Specifically, the analysis identified significant enrichment in processes such as ‘substantia nigra development’, ‘regulation of intrinsic apoptotic signalling pathway in response to osmotic stress by p53 class mediator’ and ‘positive regulation of endothelial cell chemotaxis to fibroblast growth factor’. These findings suggest that the dysregulated expression of miR-16-5p, miR-99a-5p and miR-191-5p affects neuronal development and survival, as well as modulates the inflammatory environment within the CNS. Moreover, the involvement of these miRNA targets in the ‘negative regulation of blood-brain barrier permeability’ and ‘positive regulation of AMPA receptor activity’ indicates a potential impact on synaptic plasticity and neuroprotection. The enriched biological processes also included ‘cartilage homeostasis’ and ‘regulation of branching involved in salivary gland morphogenesis by mesenchymal-epithelial signalling’, indicating that the dysregulation of miR-16-5p, miR-99a-5p and miR-191-5p also has implications beyond the CNS, possibly affecting other physiological systems and contributing to the complex multisystem nature of ALS.

Finally, we used the RNAlocate database (Cui et al., 2022) to identify the subcellular localisation of our three candidate miRNAs. Although specific localisation data for these miRNAs with regards to microglia were not available, data on other tissues and cell lines, including various CNS and immune cells, revealed a predominant association with exosomes (Table S8). This supports our hypothesis that our miRNAs of interest are indeed released into the extracellular environment and thus have the capacity of targeting genes in surrounding cells.

## DISCUSSION

Our main aim was to determine whether there is a differential release of miRNAs from transgenic microglia, in the presence or absence of LPS stimulation. LPS is a known inducer of the transcription factor nuclear factor κ-light-chain-enhancer of activated B cells (NF-κB). NF-κB regulates genes responsible for the innate and adaptive immune response, and several studies have shown that this transcription factor is upregulated in glial cells of both sporadic and patients with familial ALS (Frakes et al., 2014; Maruyama et al., 2010; Swarup et al., 2011). The use of LPS as a stimulus has further relevance in the context of ALS, as previous research has shown elevated LPS levels in the blood plasma of patients with sporadic ALS, which positively correlates with the levels of macrophage/monocyte activation (Zhang et al., 2009). In our study, stimulation of mouse microglia with LPS for 24 h successfully resulted in comparable levels of the pro-inflammatory response between males and females and among wildtype transgenic, mutant transgenic and non-transgenic microglia, in terms of the expression of two inflammatory cytokines, *Tnf* and *Il1b*, at this timepoint. This suggests that the *TARDBP^M337V^* mutation does not alter the cytokine inflammatory response of microglia immediately after 24 h of continuous exposure to LPS (at least for the tested cytokines). However, in the absence of LPS stimulation, the influence of the *TARDBP^M337V^* transgene on cytokine response appears to be complex as the baseline expression of *Tnf* was reduced by the presence of two copies of the mutant transgene in male microglia. This underscores distinct regulatory roles of TDP-43 under basal versus inflammatory conditions. At baseline, TDP-43 may regulate *Tnf* mRNA stability through interactions with RNA-binding proteins or miRNAs, a process potentially disrupted by the M337V mutation. Upon LPS stimulation, however, the activation of robust pathways such as NF-κB signalling may override these regulatory effects, maintaining *Tnf* expression levels. The absence of similar baseline *Tnf* reductions in female mutant microglia indicates sex-specific differences in TDP-43 regulation, highlighting the need for further research to elucidate these mechanisms. Therefore, the precise effects of this mutation on the cytokine response of microglia, particularly in the context of different sexes, warrants further investigation.

A number of studies have observed that the intracellular miRNA content of microglia or monocytes is altered upon activation with different stimuli or in different disease states (Falcão et al., 2017; Butovsky et al., 2012; Krasemann et al., 2017). Furthermore, inflammation-related miRNA levels increase in the serum of mice following injection of LPS, although the source of these miRNAs was not determined (Li et al., 2018). The present study complements these findings by showing that, compared to vehicle treatment, LPS stimulation resulted in a significant alteration of certain miRNAs released by microglia, particularly in females. Previous research has already shown that male and female microglia exhibit gene expression differences and morphological differences, and that their response to environmental insults is sex dependent (Dubbelaar et al., 2018; Thion et al., 2018; Hanamsagar et al., 2017). However, the present study is the first to observe sex-specific differences in terms of their released miRNAs. We also showed that the expression of the human *TARDBP^M337V^* transgene significantly affected the release of miRNAs from microglia, with some miRNAs showing a dose-dependent effect. An example of this dose-dependent effect is the release of miR-9-3p, which is higher in homozygous than in hemizygous mutant transgenic microglia. Although not followed up by RT-qPCR in our study, this miRNA is known as one of the most highly expressed miRNAs in the developing and adult vertebrate brain, with studies highlighting its importance in the regulation of neural progenitors (Coolen et al., 2013) as well as its role in the differentiation of spinal motor neurons (Otaegi et al., 2011). Additionally, this miRNA was previously found downregulated within human induced pluripotent stem cell-derived neurons carrying the *TARDBP^M337V^* mutation, underscoring cell-type-specific effects in ALS pathology (Zhang et al., 2013). Taken together, these findings raise new questions about the role of mutant microglia in ALS. It may be that despite the apparently non-inflammatory phenotype of microglia in this TDP-43 mouse model, these cells can still influence the disease via miRNA pathways, especially given the known role of TDP-43 in RNA metabolism. In general, extracellular miRNAs can be found both encapsulated within extracellular vesicles and bound to proteins. The distribution between these two forms can vary depending on the physiological and pathological contexts. In addition, extracellular vesicle-encapsulated miRNAs have the potential to function as paracrine or even endocrine gene regulators in target cells. The miRNAs identified in this study encompass all extracellular miRNAs and, therefore, further studies are required to ascertain whether these miRNAs are cargoes of extracellular vesicles and possibly to reveal novel insights into ALS pathology.

Furthermore, using RT-qPCR, we confirmed the downregulated release of miR-16-5p, miR-99a-5p and miR-191-5p by homozygous mutant transgenic microglia compared to that by non-transgenic controls or hemizygous wildtype transgenic microglia, depending on the statistical model. Our choice to focus on non-transgenic microglia as the main control was driven by the minimal differential expression observed when comparing wildtype transgenic samples to non-transgenic samples (only two miRNAs were dysregulated; Fig. 4). This suggests that the presence of the wildtype transgene itself does not account for the majority of observed differences. On the contrary, the presence of the homozygous mutant transgene dysregulated another ten miRNAs (Fig. 4), indicating a significant effect of the homozygous mutation beyond the mere presence of the transgene. Therefore, comparing the mutant transgenic samples to non-transgenic controls highlights the specific contribution of the M337V mutation to the miRNA changes. Our three validated miRNAs, miR-16-5p, miR-99a-5p and miR-191-5p, have been implicated in various biological processes, including cell proliferation, differentiation, apoptosis, cell cycle regulation and the immune response (Cimmino et al., 2005; Liu et al., 2008; Bandi and Vassella, 2011; Rissland et al., 2011; Jing et al., 2005; Sun et al., 2011, 2014; Wang et al., 2017). There is a strong link between miR-16-5p and ALS in the literature, as we and others have shown that this miRNA is downregulated in the blood (Liguori et al., 2018; Joilin et al., 2020) and cerebrospinal fluid (Waller et al., 2018) of patients with sporadic ALS, as well as in fibroblasts from patients with *C9orf72*-related ALS (Hur et al., 2023). Similarly, miR-99a-5p downregulation has also been observed in the muscle tissues from patients with ALS and the SOD1^G93A^ mouse model of ALS (Si et al., 2018), as well as in axons expressing TDP43^A315T^ and SOD1^G93A^ (Rotem et al., 2017). In light of these findings, the downregulated release of miR-16-5p and miR-99a-5p by homozygous mutant transgenic microglia may have significant implications in our understanding of ALS pathogenesis. Future studies should focus on elucidating the exact role these miRNAs play in ALS and whether modulating their levels could present a novel therapeutic approach.

We also conducted a parallel analysis for comparison purposes, utilising the same statistical procedures on the RT-qPCR data, but only including the original set of samples that were also subjected to RNA sequencing. This was carried out to assess the potential impact of sample size on the detection of the effects and interactions we were investigating. When we restricted our analysis to the original, smaller dataset, none of our results reached statistical significance. This does not necessarily discount the findings from the larger sample set, but rather highlights the sensitivity of such investigations to sample size. Inclusion of additional samples in our main analysis was intended as a strategy to increase the robustness of our findings and ensure that they were not due to chance or random variability. These results underscore the importance of robust sample sizes in the detection of nuanced effects and interactions in biological systems, and the caution that should be exercised in interpreting findings from smaller sample sets.

Nevertheless, the discrepancies observed between the sequencing and RT-qPCR results could be attributed to several factors. First, technical variation between the two methods might contribute to the inconsistencies, due to differences in the efficiency of reverse transcription, amplification and normalisation using reference miRNAs, which might have led to variations in the detected expression levels. Second, the addition of more samples in the RT-qPCR experiment may have introduced more biological variability. Finally, although RNA sequencing has a broad dynamic range, RT-qPCR can often detect small changes in gene expression that are not detected by RNA sequencing.

Our gene set enrichment analysis revealed that the dysregulated miRNAs are predicted to target genes involved in neuronal development and function. It is important to experimentally validate some of these gene targets in future studies. Crucially, not only does a microglial miRNA need to find its way towards a neuronal cell, potentially via extracellular vesicles, but its target gene(s) must also be expressed within the neuron at that specific time, for it to have any functional effects. Nevertheless, we can hypothesise that the reduced release of our two candidate miRNAs and possibly others may affect normal neuronal function. Depending on whether the affected genes are positive or negative regulators of neuronal development and activity, this may implicate these miRNAs in neurodegeneration. Although we do not provide direct evidence that miRNAs released from microglia can have such effects on neurons, previous research has shown that uptake of miRNAs released from one cell type by another cell type (Aucher et al., 2013; Mittelbrunn et al., 2011; Zhou et al., 2014; Ridder et al., 2014; Pinto et al., 2017), including from microglia to neurons (Prada et al., 2018; Huang et al., 2017; Frühbeis et al., 2013; Varcianna et al., 2019), is possible. Furthermore, in addition to their gene repressor role, extracellular miRNAs have also been shown to activate intracellular neuronal receptors, leading to neurodegeneration (Park et al., 2014; Lehmann et al., 2012) or promoting recovery following neuronal insult (Xin et al., 2013), depending on which miRNAs are delivered. As the microglia used in the current study were extracted from a mouse model of ALS, it would be interesting to investigate whether their released miRNAs can be taken up by disease-relevant cell types, such as cortical and spinal motor neurons, where they may target genes involved in neurodegeneration.

Traditionally, microglia have been implicated in neuroinflammatory diseases due to their reactive phenotype, which is primarily characterised by cytokine release and phagocytosis, and several attempts have been made to develop treatments that modulate these processes. However, this study indicates that microglia should also be considered as possible regulators of gene silencing in other cell types via their miRNA release, thus opening new avenues in neuroinflammation research. In addition, TDP-43 mutations may further modulate this microglial response and thus have further implications in ALS research. Future studies could also examine whether other ALS-linked mutations, particularly mutations in genes involved in RNA metabolism, such as *FUS*, can similarly affect microglia-derived miRNAs. Moreover, an important question arising from this study is whether the same miRNAs that were dysregulated in the microglial culture medium are similarly dysregulated within the microglia themselves. If so, it may suggest a passive and random release of these miRNAs by the cells, whereas a different intracellular miRNA profile may suggest an active sorting mechanism of a selection of miRNAs that are specifically destined for release. In fact, such an active sorting of miRNAs into exosomes has already been observed in HEK293 cells (Guduric-Fuchs et al., 2012), and it would be interesting to examine this mechanism in microglia in the future. Finally, considering the observed sex differences in ALS incidence and prevalence (Manjaly et al., 2010; McCombe and Henderson, 2010), our findings of more pronounced dysregulation of extracellular microglial miRNAs in females further underscore the importance of investigating sex differences in ALS research. Future analyses on existing datasets to uncover sex-specific differences in miRNA profiles could be highly informative. This approach could uncover sex-specific biomarkers or pathways that are differentially affected in ALS, leading to insights into why the disease progression or response to treatment may differ between sexes.

## MATERIALS AND METHODS

### Animals

Procedures involving animals were approved by the UK Home Office under the UK Animals Scientific Procedures Act 1986 and the University of Sussex Animal Welfare and Ethics Review Board. Mice (*Mus musculus*) were kept on a 12 h light/12 h dark cycle (lights on at 07:00) in cages with up to five littermates and free access to food and water. Littermates were used wherever possible. The sex of all neonatal mice was determined by simplex PCR as previously described (Tunster, 2017). Transgenic mice carrying the human *TARDBP* gene (either wildtype or with the M337V mutation; The Jackson Laboratory strain 029266) were bred with C57BL/6J mice (The Jackson Laboratory strain 000664) to create in-house colonies. Generation of these transgenic mice is described elsewhere (Gordon et al., 2019). Briefly, a bacterial artificial chromosome carrying the human *TARDBP* locus was integrated into the *Rosa26* locus of mouse embryonic stem cells, which were then used to produce transgenic mice on a C57BL/6 background (Gordon et al., 2019). Six male and six female non-transgenic (*TARDBP^−/−^*), hemizygous wildtype (*TARDBP^−/+^*), hemizygous mutant (*TARDBP^−/M337V^*) and homozygous mutant (*TARDBP^M337V/M337V^*) transgenic mice per genotype were used for primary mixed glial cell cultures. Due to difficulties encountered with the breeding and maintenance of homozygous wildtype (*TARDBP^+/+^*) transgenic mice (i.e. unexpectedly high number of unsuccessful breeders and overrepresentation of *TARDBP*^+/+^ mice that died on the first day after birth), the required numbers of homozygous *TARDBP*^+/+^ mice were not obtained; therefore, they were not included in the study. We suspect that these challenges may stem from a genetic drift or an oversight during the maintenance of the wildtype transgenic colony, which may have inadvertently impacted pup survival. Nevertheless, the hemizygous *TARDBP^−/+^* bred normally and were thus included in the study. We advise readers to interpret findings involving the hemizygous wildtype transgenics with caution, considering the potential implications of any breeding anomalies on the generalisability of the results.

### Coating of tissue culture surfaces

Cell culture flasks (75 cm^2^; Thermo Fisher Scientific, 15350591) were coated with 7 ml of poly-D-lysine (10 μg ml^−1^ in molecular biology-grade water) for 2 h at 37°C. The flasks were then washed three times with 7 ml of molecular biology-grade water and left to air-dry completely before use.

### Dissection and culture of primary mixed glia

Mixed glia were cultured as previously described (Lian et al., 2016) with some modifications. Briefly, the mice were sacrificed by cervical dislocation, followed by exsanguination at postnatal days 0-3, and the brains were removed and kept immersed in dissection medium made up of Hank's Balanced Salt Solution (HBSS++; Thermo Fisher Scientific, Gibco, 24020117) containing 0.6% (w/v) D-glucose (Sigma-Aldrich, G-6152), 1% (v/v) of 1 M HEPES (Sigma-Aldrich, H-4034) at pH 7.2-7.5 and 1% (v/v) penicillin/streptomycin (Thermo Fisher Scientific, Gibco, 15140122). Following removal of the meninges, the hippocampal and cortical tissues were minced using spring scissors, transferred in a tube containing 30 ml cold dissection medium and 1.5 ml of 2.5% trypsin (Thermo Fisher Scientific, Gibco, 27250018), and incubated in a 37°C water bath for 15 min with frequent swirling to dissociate the cells. Then, 1.2 ml of 1 mg ml^−1^ trypsin inhibitor [Sigma-Aldrich, T6522-25MG; suspended in Dulbecco's phosphate-buffered saline (DPBS; Sigma-Aldrich, D8537-500ML)] was added to inhibit trypsin activity, and the tube was left to incubate for 1 min at room temperature. After that, 750 μl of 1.2 mg ml^−1^ DNase I (Sigma-Aldrich, DN25-100MG; suspended in HBSS++) was added to digest the sticky DNA released from dead cells, and the tube was centrifuged at 400 ***g*** for 5 min to pellet the cells. The supernatant was removed, the pellet was triturated in 5 ml pre-warmed culture medium containing 89% Dulbecco's modified Eagle medium (DMEM)/F-12 (Thermo Fisher Scientific, Gibco, 11580546), 10% heat-inactivated fetal bovine serum (Thermo Fisher Scientific, Gibco, 10270106), and 1% penicillin/streptomycin, and centrifuged again at 400 ***g*** for 5 min. The supernatant was removed and the pellet was resuspended in 5 ml pre-warmed culture medium. The cell suspension was cultured in a poly-D-lysine-coated flask with a total of 15 ml pre-warmed culture medium and incubated at 37°C and 5% CO_2_. The medium was replaced after 24 h to remove cell debris. This culture consisted primarily of astrocytes and microglia, as identified by immunocytochemistry (Fig. S4).

### Splitting flasks of mixed glia

After 7 days in culture, each flask of mixed glia was split into two new flasks, to maximise the number of cells obtained per animal while minimising the number of animals used, in accordance with the ‘Replacement, Reduction and Refinement’ (3R) principles of animal research. The culture medium was removed from the flask and the cells were washed with 7 ml pre-warmed DPBS. Then, 2 ml of pre-warmed 0.25% trypsin-EDTA (Thermo Fisher Scientific, Gibco, 11560626) was added to the cells and the flask was incubated for 5 min at 37°C and 5% CO_2_. After that, the flask was lightly tapped by hand to detach the cells and 5 ml of pre-warmed culture medium was added to the flask to inactivate the trypsin. The medium with the cells was collected into a universal tube and centrifuged at 390 ***g*** for 5 min to pellet the cells. The supernatant was removed and the cells were resuspended with a P1000 micropipette in 5 ml of pre-warmed culture medium. The cell suspension was equally distributed into two new flasks and the culture medium was topped up to 15 ml per flask. The flasks were then returned to 37°C and 5% CO_2_ for 14 days. Every 4 to 5 days, the culture medium of each flask was replaced. After approximately 7 days in culture, the flasks became 100% confluent, with a monolayer of astrocytes forming and microglia growing at the top and bottom of this monolayer.

### Isolation of microglia from mixed glial cultures

To remove astrocytes from the mixed cultures, a mild trypsinisation method resulting in >98% pure microglia cultures was used, as previously described (Saura et al., 2003), with some modifications. Briefly, the culture medium was removed and the cells were washed with 7 ml pre-warmed DPBS. The mixed glia-conditioned medium was passed through a 0.2 μm filter to remove any cells and was kept for later. Then, 7 ml of 0.25% trypsin-EDTA diluted 1:4 in DMEM/F-12 was added to the cells and the flasks were incubated at 37°C and 5% CO_2_ for ∼1.5 h, until the astrocyte monolayer and the microglia growing on top of it completely detached. The remaining, attached cells were only microglia. After that, 7 ml of pre-warmed culture medium was added to the flask to inactivate the trypsin, and the entire medium with the floating cells was discarded. The remaining microglial cells were washed twice with 7 ml pre-warmed DPBS to ensure the removal of astrocytes. Then, 4 ml of 0.25% trypsin-EDTA was added to the flask and the cells were incubated for 5 min at 37°C, 5% CO_2_ to detach them. After incubation, the cells were scraped off the bottom of the flask using a cell scraper and 8 ml of pre-warmed culture medium was added to inactivate the trypsin. The cell suspensions from both flasks for each animal were collected into a single tube and centrifuged for 10 min at 390 ***g*** to pellet the cells. The supernatant was removed and the cells were resuspended in 2 ml of astrocyte-conditioned medium (a mixture of 50% fresh culture medium and 50% mixed glia-conditioned culture medium collected at the beginning of the isolation). The cells were counted with a haemocytometer, plated at 100,000 cells/well in 24-well plates (1 ml/well), using 50% fresh and 50% conditioned medium, and incubated at 37°C and 5% CO_2_ for 48 h. The purity of the microglia cultures was visually estimated to be >98%, based on the morphological characteristics of the two major cell types – astrocytes and microglia – observed under a phase-contrast microscope (Fig. S5).

### LPS stimulation of microglia in culture

Forty-eight hours following plating of the microglia, the culture medium was discarded, the cells were washed with 1 ml/well of pre-warmed DPBS to ensure complete removal of the remaining serum, and 1 ml/well of pre-warmed serum-free culture medium was added to the cells for a further 24 h. This medium change was necessary to remove any molecules released by the cells, which may be triggered by the isolation procedure or induced by the attachment to the plate, as these could potentially influence the inflammatory state of the microglia. Furthermore, serum may contain proteins that act as cofactors for various agonists and activate undesired receptor systems, which could lead to responses unrelated to what is being measured, so its removal is necessary to avoid this. Importantly, serum has been shown to contain small ncRNA contaminants (Mannerström et al., 2019), which could confound the detection of microglia-derived ncRNAs in subsequent experiments.

Following a rest period of 24 h in serum-free culture medium, the cells were treated with fresh serum-free culture medium containing either 250 ng ml^−1^ LPS (Sigma-Aldrich, L2654-1MG) or vehicle (HBSS) for a further 24 h. The LPS concentration and incubation time were selected based on optimisation experiments showing that 24 h are sufficient to substantially stimulate the cells without significant cytotoxicity at this concentration (Fig. S2A,B). Furthermore, optimisation experiments also suggested that 24 h is a sufficient duration for extracellular miRNAs to accumulate into the culture medium in the absence of any stimulation (Fig. S2C,D). After stimulation, the culture medium was collected and immediately frozen at −80°C. The cells were washed with 1 ml pre-warmed DPBS per well; then, 260 μl of buffer RLT (QIAGEN, 79216) containing 1% β-mercaptoethanol (Thermo Fisher Scientific, M/P200/05) was added to the cells to detach and lyse them. The cell suspension was collected into a tube and immediately vortexed for 1 min to homogenise the cells, then snap-frozen in liquid nitrogen, followed by long-term storage at −80°C until RNA extraction.

### Intracellular RNA extraction, cDNA synthesis and RT-qPCR

The total RNA from the collected microglia was extracted using the miRNeasy Tissue/Cells Advanced Mini Kit (QIAGEN, 217604), according to the manufacturer's protocol. The amount of RNA was quantified using the Qubit RNA high-sensitivity assay kit (Thermo Fisher Scientific, Q32854) with the Qubit 3.0 fluorometer (Life Technologies, Q33216). Complementary DNA (cDNA) synthesis using 8 ng of total RNA was carried out using the High-Capacity RNA-to-cDNA kit (Thermo Fisher Scientific, 4388950), according to the manufacturer's protocol. A 10-fold dilution of the cDNA was then used for SYBR Green RT-qPCR to quantify the expression levels of *Tnf* and *Il1b* (normalised to the expression levels of *Gapdh* and *Pgk1*), using the SYBR qPCR 2× mix (AptoGen, 411101.5.1A25) and the following primers (5′-to-3′ sequence): *Tnf* forward primer, 5′-GGTGCCTATGTCTCAGCCTCTT-3′; *Tnf* reverse primer, 5′-GCCATAGAACTGATGAGAGGGAG-3′; *Il1b* forward primer, 5′-TGCCACCTTTTGACAGTGATG-3′; *Il1b* reverse primer, 5′-TGATGTGCTGCTGCGAGATT-3′; *Gapdh* forward primer, 5′-GGTGAAGGTCGGTGTGAACG-3′; *Gapdh* reverse primer, 5′-CAATCTCCACTTTGCCACTGC-3′; *Pgk1* forward primer, 5′-TTGTGCATTGTAGAGGGCGT-3′; and *Pgk1* reverse primer, 5′-TGACGAAGCTAACCAGAGGC-3′.

### Extracellular miRNA extraction, miRNA library preparation, Illumina sequencing, cDNA synthesis and TaqMan advanced miRNA assays

Extracellular RNA was extracted from 200 μl of microglia-conditioned culture medium using the miRNeasy Serum/Plasma Advanced Kit (QIAGEN, 217204). The QIAseq miRNA Library Kit (QIAGEN, 331505) was used with 5 μl of extracellular RNA to prepare 48 miRNA libraries for next-generation sequencing according to the manufacturer's protocol, but with the following modifications: (1) the 3′ adapter and the primer for reverse transcription were used at a 1:20 dilution, (2) the 5′ adapter was used at a 1:10 dilution, (3) all bead-washing steps were done using 500 μl of 80% ethanol instead of the recommended 200 μl, and (4) at the library amplification step, denaturation, annealing and extension were performed for 24 cycles. The tube indices from the QIAseq miRNA NGS 48 Index IL (QIAGEN, 331595) were used for library preparation.

Pre-sequencing quality control of the miRNA libraries was done on an Agilent Bioanalyzer using a high-sensitivity DNA chip (Agilent Technologies, 5067-4626). Then, the Qubit dsDNA Assay Kit (Thermo Fisher Scientific, Q32854) was used with the Qubit 3.0 fluorometer to quantify the amount of library, before diluting and pooling the samples at equimolar concentrations for sequencing. The sequencing of the miRNA libraries was performed by the next-generation sequencing facility at the University of Leeds, where the BluePippin system (Sage Science, BLU0001) was used to size select for the libraries between 170 and 200 bp to extract miRNA and piRNA libraries. The Bioanalyzer and Qubit 3.0 fluorometer were then used to confirm the presence of correctly sized libraries and quantify the amounts. The libraries were then prepared for 100 bp single-end sequencing using the P3 kit on the Illumina NextSeq 2000, with each sample split over two lanes on a flow cell. Upon completion of the sequencing, the sequencing facility performed the basecalling and trimmed the 5′ adapters from the reads.

Furthermore, 2 μl of extracellular RNA were used with the TaqMan Advanced miRNA cDNA Synthesis Kit (Thermo Fisher Scientific, A28007). The miRNAs with the least expression variability among the 48 sequenced samples were identified using geNorm (Vandesompele et al., 2002) in R version 4.2.0 (https://www.R-project.org/), and the three least variable miRNAs were selected as normalisers for RT-qPCR validation. Specific miRNAs of interest were then used with TaqMan Advanced miRNA assays (Thermo Fisher Scientific, 15412184) to validate the sequencing results by RT-qPCR, according to the manufacturer's protocol, using fast cycling and with the qPCR Lo-ROX 2× mix (AptoGen, 412101.5). The TaqMan Advanced miRNA assays for the normaliser miRNAs were mmu-miR-28a-3p (mmu481665_mir), mmu-miR-32-5p (mmu482950_mir) and mmu-miR-190a-5p (mmu481335_mir), and those for the target miRNAs were mmu-miR-16-5p (mmu482960_mir), mmu-miR-29b-3p (mmu481300_mir), mmu-miR-99a-5p (mmu478519_mir) and mmu-miR-191-5p (mmu481584_mir).

### Bioinformatic analysis

Quality control of the sequencing data was undertaken using the FastQC tool (http://www.bioinformatics.babraham.ac.uk/projects/fastqc/) via the Galaxy webserver (Afgan et al., 2018). Primary miRNA quantification was performed using predefined analysis pipelines via the QIAGEN RNA-seq Analysis Portal 2.0 (available at https://geneglobe.qiagen.com/us/analyze). The reads were aligned to the latest mouse genome assembly available at the time of analysis (GRCm38.p6; RefSeq assembly accession GCF_000001635.26) using Bowtie (Langmead et al., 2009). This alignment strategy mapped the reads to those available in miRBase v21 (Kozomara et al., 2019). Secondary analysis to calculate differential miRNA expression between the different samples was undertaken in R version 4.2.0 using the DESeq2 package (release 3.15) (Love et al., 2014). We designed our analysis to consider both the effects of genotype and treatment on miRNA expression, as well as the potential interaction between these two factors. Specifically, our design formula was ∼Mouse+Genotype+Treatment+Genotype:Treatment. The ‘Mouse’ term in the model accounts for the pairing of samples from each biological replicate, adjusting for their inherent non-independence due to one sample receiving LPS treatment and the other receiving the vehicle treatment. Sex was excluded from the model to maintain simplicity and avoid overfitting. Therefore, the model was applied to each sex separately to address potential sex-specific effects. We then performed a series of contrasts to understand the different effects in our experiment. Firstly, the effect of treatment was assessed by comparing miRNA expression between the LPS and vehicle conditions. In other words, this comparison answers the question: how does the miRNA expression for the LPS treatment compare to that for the vehicle treatment, after controlling for the genotype effect?. Secondly, to examine the effect of different genotypes, we performed three comparisons: (1) *TARDBP^M337V/M337V^* versus *TARDBP^−/−^*, (2) *TARDBP^−/M337V^* versus *TARDBP^−/−^*, and (3) *TARDBP^−/+^* versus *TARDBP^−/−^*. In other words, each of these comparisons answers the question: how does the miRNA expression in genotype X compare to that in the control (*TARDBP^−/−^*) genotype, after controlling for the treatment effect?. Finally, we performed pairwise contrasts of the interaction terms to understand how the response to LPS treatment varies between different genotypes. Specifically, we examined the differences between (1) *TARDBP^M337V/M337V^* and *TARDBP^−/−^*, (2) *TARDBP^M337V/M337V^* and *TARDBP^−/M337V^*, (3) *TARDBP^M337V/M337V^* and *TARDBP^−/+^*, (4) *TARDBP^−/M337V^* and *TARDBP^−/+^*, (5) *TARDBP^−/M337V^* and *TARDBP^−/−^*, and (6) *TARDBP^−/+^* and *TARDBP^−/−^*. In other words, each of these comparisons answers the question: how does the difference in miRNA expression between LPS and vehicle treatments for genotype X compare to that same difference for genotype Y?. Any miRNAs with an FDR <10% were considered to be significantly dysregulated.

Target prediction of candidate miRNAs was performed using TargetScan version 8.0 (Agarwal et al., 2015) and miRDB version 6.0 (Chen and Wang, 2020; Liu and Wang, 2019). Additionally, miRTargetLink version 2.0 (Kern et al., 2021) and miRTarBase version 9.0 (Huang et al., 2022) were used to identify experimentally validated target genes of our RT-qPCR-validated candidate miRNAs. Gene set enrichment analysis of target genes was carried out using Gene Ontology release 17 January 2024 (Ashburner et al., 2000; The Gene Ontology Consortium et al., 2023). RNA localisation analysis was performed using RNAlocate version 2.0 (Cui et al., 2022).

### Statistical analysis

RT-qPCR data were analysed in GraphPad Prism version 10.0.0. Outliers were identified by the ROUT test with Q=1% and were removed before further analysis, as indicated in the figure legends. Data on *Tnf* and *Il1b* mRNA expression were analysed using a two-way repeated-measures ANOVA with Šidák's post hoc test. Data on miRNA expression were analysed with either paired two-tailed *t*-tests or with two-way repeated-measures ANOVA, as indicated in the figure legends. The threshold for significance was set to 5%. Where statistically significant main effects were identified by ANOVA, post hoc analysis was performed using the two-stage step-up method of Benjamini, Krieger and Yekutieli to correct for multiple comparisons by controlling the FDR (set to 5%).

## Supplementary Material

10.1242/dmm.050638_sup1 Supplementary information

Table S1. Dysregulated release of miRNAs upon LPS stimulation, after adjusting for genotype effects.

Table S2. Dysregulated release of miRNAs in female transgenic samples, after adjusting for treatment effects.

Table S3. Interaction between genotype and treatment in dysregulating release of miRNAs.

Table S4. Overlap between different genotypes in terms of the differences in the response to LPS treatment, compared to vehicle treatment.

Table S5. Predicted targets of mmu-miR-16-5p, mmu-miR-99a-5p, and mmu-miR-191-5p using miRDB and TargetScan.

Table S6. Validated targets of mmu-miR-16-5p, mmu-miR-99a-5p, and mmu-miR-191-5p from miRTarBase and miRTargetLink.

Table S7. Gene Ontology biological processes significantly enriched in the list of predicted and validated targets of mmu-miR-16-5p, mmu-miR-99a-5p, and mmu-miR-191-5p.

Table S8. RNAlocate results showing the localisation of mmu-miR-16-5p, mmu-miR-99a- 5p, and mmu-miR-191-5p primarily in exosomes.
