# Supplementary material for: An ALS-associated mutation dysregulates microglia-derived extracellular microRNAs in a sex-specific manner
Source: Dis Model Mech. 2024 May 29;17(5):dmm050638. doi: 10.1242/dmm.050638 (PMC11152562; doi:10.1242/dmm.050638)
Supplement: Supplementary information [file dmm-17-050638-s1.pdf]

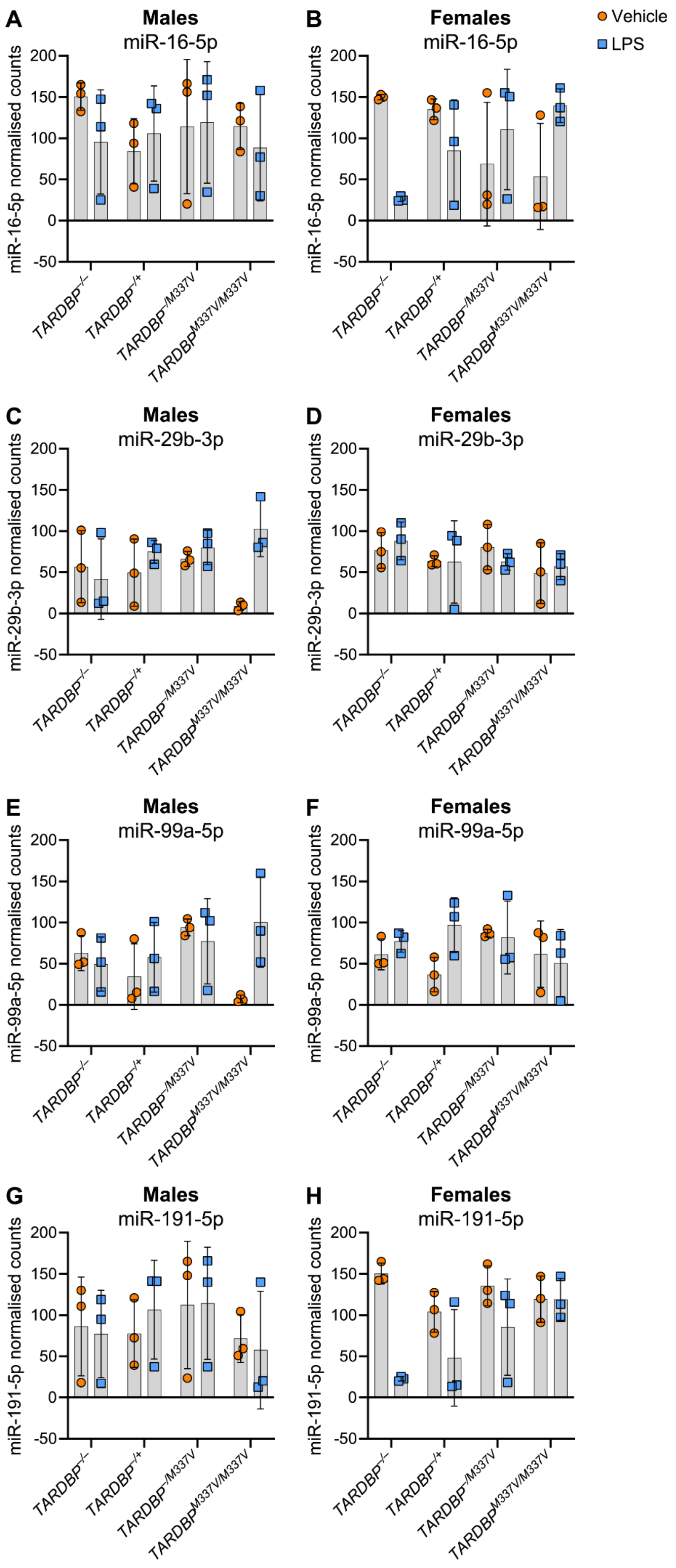

Disease Models & Mechanisms • Supplementary information

Fig. S1. Normalised counts used by DESeq2 for analysis, for the four candidate miRNAs selected for RT-qPCR validation. (**A-B**) Normalised counts for miR-16-5p. (**C-D**) Normalised counts for miR-29b-3p. (**E-F**) Normalised counts for miR-99a-5p. (**G-H**) Normalised counts for miR-191-5p. Data is shown as mean±s.d. $N = 3$ biological replicates per genotype per sex.

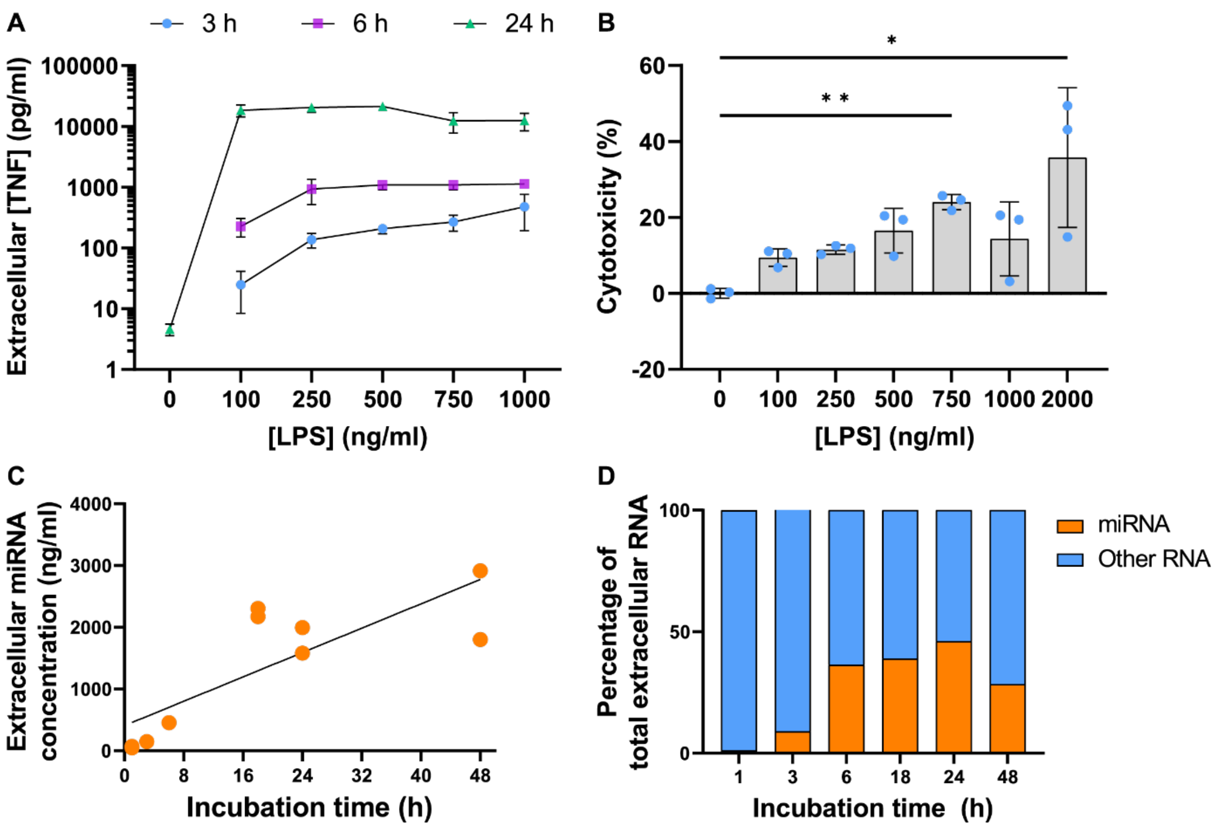

Fig. S2. Optimisation experiments for the selection of the appropriate concentration of LPS and incubation time with LPS.

(**A**) Effect of increasing LPS concentrations and incubation times on TNF release by primary microglia, measured by an ELISA. N = 3 biological replicates per condition. (**B**) Cytotoxicity of increasing LPS concentrations on primary microglia after a 24-hour incubation time. LPS concentrations above 250 ng/ml result in >15% cytotoxicity. N = 3 biological replicates per condition. Kruskal-Wallis test with Dunn's post-hoc. *P<0.05, **P<0.01. (**C**) Correlation between extracellular miRNA concentration and incubation time in serum-free culture medium. The concentration of extracellular miRNA increases with increasing incubation time. (**D**) The proportion of total extracellular RNA that is miRNA at different incubation times. N = 1-2 biological replicates per incubation time. The concentration of extracellular miRNA decreases beyond 24 hours of incubation time.

Disease Models & Mechanisms • Supplementary information

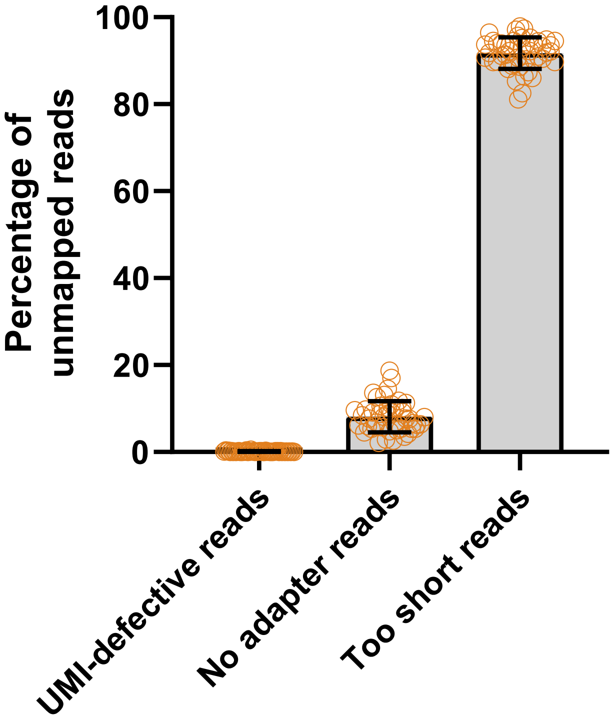

Fig. S3. Percentage of reads that did not map to the genome. Most unmapped reads did not map due to being too short. Data is shown as mean±s.d from 48 miRNA libraries.

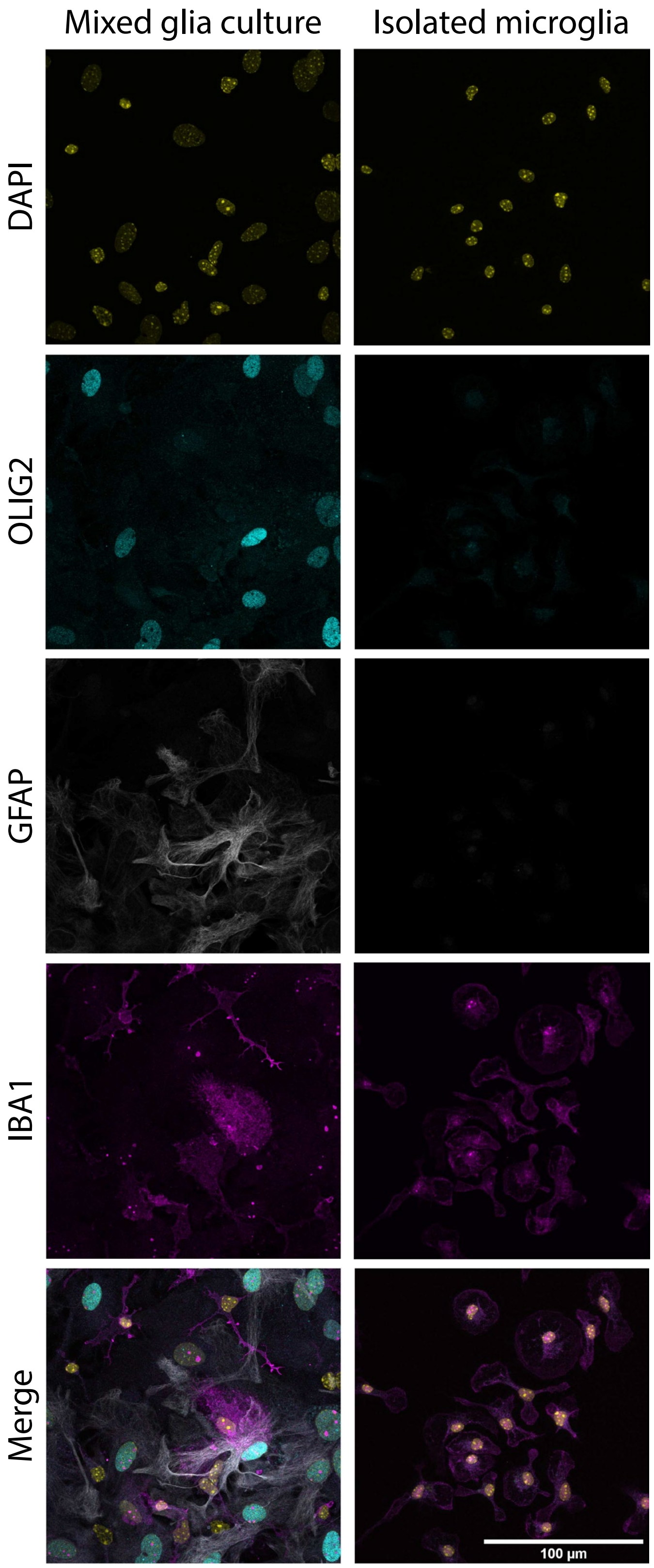

Fig. S4. Immunocytochemical characterisation of the cell populations present in mixed glial cultures. Representative images showing the presence of microglia (IBA1^{+}), astrocytes (GFAP^{+}), oligodendrocytes (OLIG2^{+}), and radial glia (GFAP^{+} plus OLIG2^{+}) in the mixed cultures, as expected (left column). Following the isolation procedure to remove the astrocyte monolayer and any cells on top of it, the remaining cells were pure microglia, labelled only by IBA1 (right column). Scale bar = 100 µm.

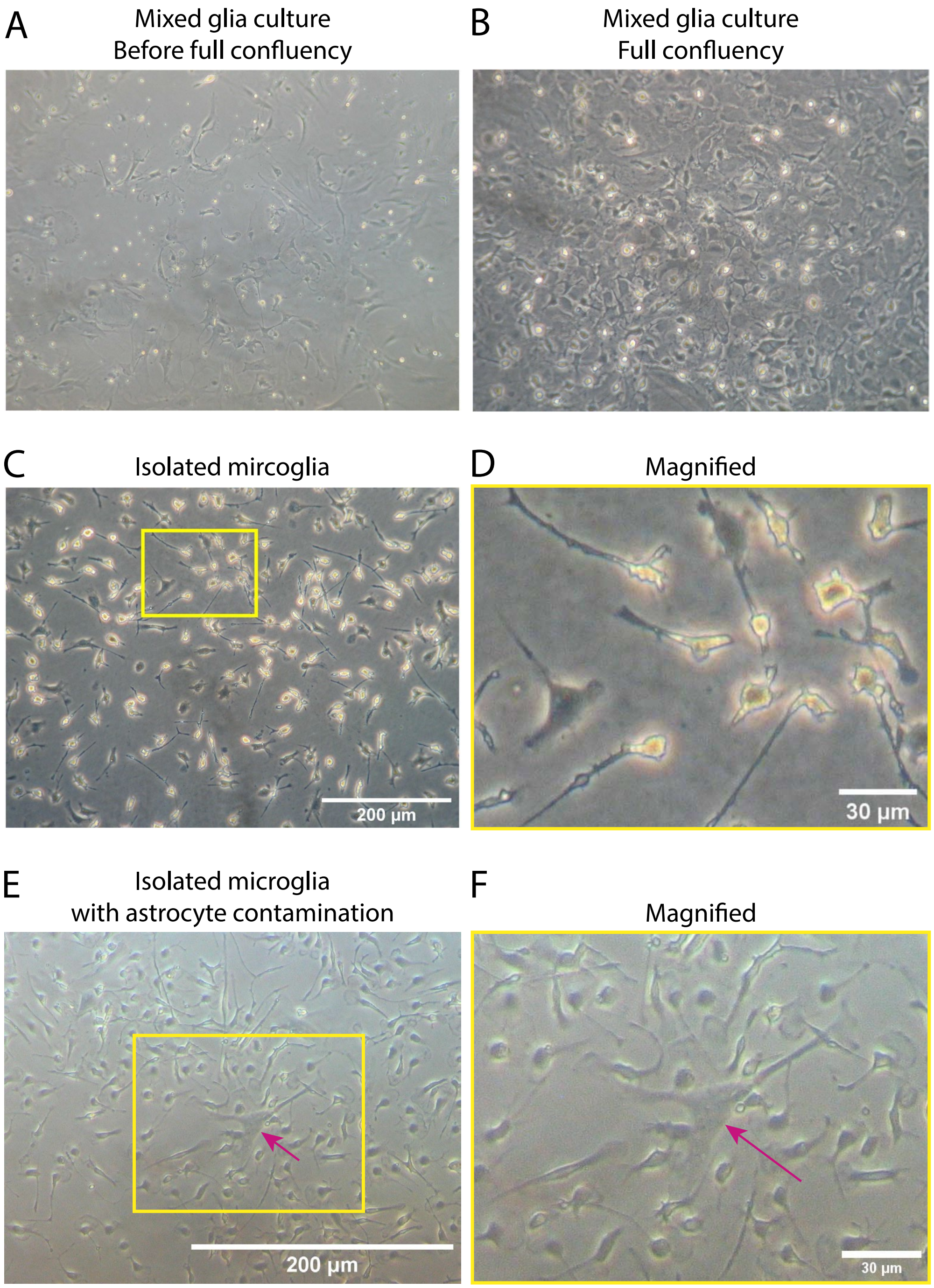

Fig. S5. Phase-contrast images showing the cell populations present in mixed glial cultures. (**A-B**) Representative images showing the mixed glial culture before and after full confluency. At full confluency, the astrocytes form a monolayer, with microglia attaching on top of it. There are also microglia attached to the bottom of the flask, below the astrocyte monolayer (not visible here). (**C-D**) Cells isolated from the bottom layer of the mixed culture are pure microglia, which begin ramifying soon after seeding, typically characterised by one or two long processes. (**E-F**) Occasionally, astrocyte contamination (arrows) in the isolated microglia was present, identified based on morphological characteristics. Astrocytes consisted of <2% of the cell population after the isolation procedure (estimated visually). Scale bar = 200 µm, or 30 µm after magnification.

Table S1. Dysregulated release of miRNAs upon LPS stimulation, after adjusting for genotype effects.

Available for download at
https://journals.biologists.com/dmm/article-lookup/doi/10.1242/dmm.050638#supplementary-data

Table S2. Dysregulated release of miRNAs in female transgenic samples, after adjusting for treatment effects.

Available for download at
https://journals.biologists.com/dmm/article-lookup/doi/10.1242/dmm.050638#supplementary-data

Table S3. Interaction between genotype and treatment in dysregulating release of miRNAs.

Available for download at
https://journals.biologists.com/dmm/article-lookup/doi/10.1242/dmm.050638#supplementary-data

Table S4. Overlap between different genotypes in terms of the differences in the response to LPS treatment, compared to vehicle treatment.

Available for download at
https://journals.biologists.com/dmm/article-lookup/doi/10.1242/dmm.050638#supplementary-data

Table S5. Predicted targets of mmu-miR-16-5p, mmu-miR-99a-5p, and mmu-miR-191-5p using miRDB and TargetScan.

Available for download at
https://journals.biologists.com/dmm/article-lookup/doi/10.1242/dmm.050638#supplementary-data

Table S6. Validated targets of mmu-miR-16-5p, mmu-miR-99a-5p, and mmu-miR-191-5p from miRTarBase and miRTargetLink.

Available for download at
https://journals.biologists.com/dmm/article-lookup/doi/10.1242/dmm.050638#supplementary-data

Table S7. Gene Ontology biological processes significantly enriched in the list of predicted and validated targets of mmu-miR-16-5p, mmu-miR-99a-5p, and mmu-miR-191-5p.

Available for download at
https://journals.biologists.com/dmm/article-lookup/doi/10.1242/dmm.050638#supplementary-data

Table S8. RNAlocate results showing the localisation of mmu-miR-16-5p, mmu-miR-99a- 5p, and mmu-miR-191-5p primarily in exosomes.

Available for download at
https://journals.biologists.com/dmm/article-lookup/doi/10.1242/dmm.050638#supplementary-data
